# Heterologous production of a plant biostimulant in *Streptomyces albidoflavus*

**DOI:** 10.1007/s00253-026-13915-w

**Published:** 2026-06-20

**Authors:** Renata Sigrist, Te Chen, Marta Reventós Montané, Peter Gockel, Yijun Qiao, Mathias Jönsson, Zhijie Yang, Tilmann Weber, Ling Ding, Emre Özdemir, Lei Yang

**Affiliations:** 1 Present Address: The Novo Nordisk Foundation Biotechnology Research Institute for the Green Transition, Technical University of Denmark, 2800 Kongens Lyngby, Denmark; 2 Present Address: Department of Biotechnology and Biomedicine, Technical University of Denmark, 2800 Kongens Lyngby, Denmark; 3 Present Address: Department of Microbiology and Immunology, The Peter Doherty Institute for Infection and Immunity, University of Melbourne, Melbourne, VIC Australia; 4 The Novo Nordisk Foundation Center for Biosustainability, Technical University of Denmark, 2800 Kongens Lyngby, Denmark

**Keywords:** *Streptomyces*, Natural products, Pteridic acids, Pathway engineering, Genome‑scale Metabolic Models (GEMs)

## Abstract

**Abstract:**

Climate change–associated abiotic stresses threaten agricultural productivity, creating a need for sustainable strategies that improve plant resilience. Pteridic acids F and H (PTA-F and PTA-H), originally isolated from *Streptomyces iranensis* HM 35, are plant growth–promoting polyketides with reported activity under drought and salinity stress. However, reported production was extremely low (~ 0.08 and 0.02 mg/L), limiting further development and application. Here, we established a heterologous production platform for PTA biosynthesis by cloning the 68-kb type I polyketide synthase biosynthetic gene cluster using Cas12a-assisted precise targeted cloning using in vivo Cre-lox recombination (CAPTURE), followed by CRISPR-Cas9-mediated genomic integration and promoter engineering in *Streptomyces* hosts. Initial heterologous expression resulted in detectable elaiophylin production but not PTA, whereas BGC engineering with the strong constitutive *kasO*p* promoter enabled PTA production (although below the limit of quantification). Genome-scale metabolic model–guided media optimization further improved production and fed-batch fermentation yielded 1.7 mg/L PTA in J1074-PTA-*kasO*p* and 2.8 mg/L PTA in NBC1270-PTA-*kasO*p*. These titers represent a more tha n 20-fold increase compared with the native producer under comparable conditions. This work provides the first functional heterologous platform for PTA biosynthesis and demonstrates how synthetic biology and genome-scale metabolic modeling can be combined to improve production of complex plant-beneficial polyketides.

**Key points:**

• *Direct BGC cloning and engineering enabled production of PTA in heterologous host.*

• *Genome-scale metabolic models (GEMs) guided media optimization for PTA production.*

• *Fed-batch fermentation achieved* > *20-fold PTA titer improvement over native strain.*

**Supplementary Information:**

The online version contains supplementary material available at https://doi.org/10.1007/s00253-026-13915-w.

## Introduction

Climate change can severely affect food security by disrupting agriculture through rising temperatures, shifting rainfall patterns, and extreme weather. Water scarcity and increased soil salinity, especially in coastal areas, further reduce crop productivity and thus adaptation is essential to ensure stable food systems (Wheeler and von Braun 2013).

Plant abiotic stress can be modulated by using genetically modified crops, small-molecule chemical treatments, bioremediation or biostimulation (Kallifidas et al. 2018; Zhang et al. 2022). *Streptomyces* are well known as part of soil microbial communities having an important role as plant protector or growth-promoter (Nazari et al. 2023). Natural products produced by these bacteria can be a more sustainable solution to address problems such as drought and salinity stress (Viaene et al. 2016; Fitzpatrick et al. 2018; Nazari et al. 2023).

**Fig. 1:**
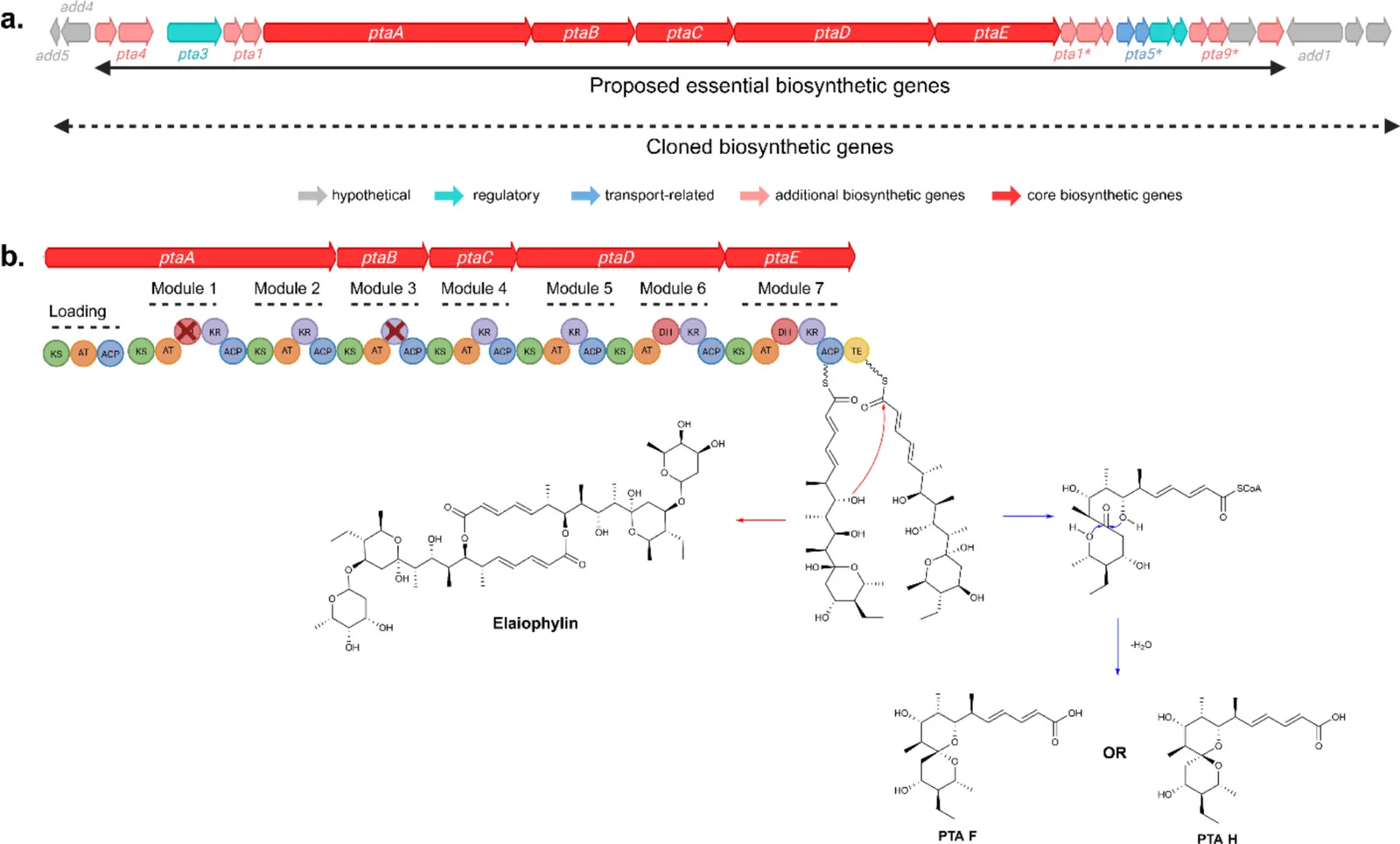
Pteridic acids and elaiophylin biosynthetic pathway. **a **Proposed essential biosynthetic genes and targeted region for CAPTURE cloning (~ 68 kb). **b **A type I modular polyketide synthase (PKS) is responsible for PTA and elaiophylin biosynthesis. Core biosynthetic genes (*pta*A – E) consist of one loading module and seven extender modules, each responsible for the incorporation of one acyl-CoA unit to the polyketide chain. Modules 1 and 3 contain two inactive domains, dehydratase (DH) and ketoreductase (KR), respectively. Final module 7 is responsible for polyketide chain release. Elaiophylin is formed by dimerization of two polyketide chains in TE domain – biosynthetic route highlighted with red arrow. Biosynthetic route highlighted with blue arrow leads to PTA derivatives. *Created in BioRender. Sigrist, R. (2026) *https://BioRender.com/de8od6a

*Streptomyces iranensis* HM 35 has been reported to alleviate drought and salinity stress in plants through the production of the isomers pteridic acid F and H (PTA-F and H) (Yang et al. 2023). These compounds are synthesized by a type I modular polyketide synthase (PKS) encoded within a biosynthetic gene cluster (BGC), hereafter referred to as PTA BGC, which also directs the biosynthesis of the related macrolide elaiophylin (Fig. 1) (Yang et al. 2023).

Type I PKSs assemble complex polyketide structures through the sequential condensation of acyl-CoA extender units, such as malonyl-CoA, methylmalonyl-CoA and ethylmalonyl-CoA in PTA biosynthesis. Following assembly, additional tailoring enzymes encoded within the BGC are expected to introduce post-PKS modifications, such as oxidation, reduction, and cyclization reactions, resulting in structurally diverse metabolites. While both PTA and elaiophylin originate from the same PKS assembly line, they diverge in the final biosynthetic step in the TE domain: elaiophylin is proposed to arise from the condensation of two polyketide chains, whereas PTA is thought to result from the release of a single chain (Fig. 1b). However, the precise mechanism underlying this divergence remains unclear (Yang et al. 2023).

In addition to this biosynthetic complexity, the application of *S. iranensis* HM 35 in agriculture is constrained by several factors. These include the very low production titers of PTA (~ 0.08 mg/L PTA F; 0.02 mg/L PTA H), the presence of multiple co-produced bioactive secondary metabolites such as azalomycin (Cheng 2010), nigericin (Sahu et al. 2020), rapamycin (Schwecke et al. 1995), hygrocin (Li et al. 2014) and alligamycin (Yang et al. 2024) and the limited availability of genetic tools and robustness of the native strain for industrial fermentation processes.

Alternatively, heterologous expression bypasses the need to develop strain-specific genetic tools and enables functional analysis of BGCs from both cultured and uncultured microbes, facilitating rapid pathway characterization, genetic manipulation, and universal platform optimization for natural products production (Zhang et al. 2019; Kang and Kim 2021; Kadjo and Eustáquio 2023). In this context, *S. albidoflavus* J1074 is a widely used strain for natural products heterologous expression (Lasch et al. 2026) due to its relatively small genome (6.8 Mb) (Zaburannyi et al. 2014), which is smaller than ~ 94.6% of other *Streptomyces* species (Mohite et al. 2025). It is fast growth (Zaburannyi et al. 2014) and feasible for genetic manipulation, as it is a *Sal*I (*Sal*GI) restriction-modification system mutant (Rodicio and Chater 1988). In this study, we have selected *Streptomyces albidoflavus* J1074 and *Streptomyces albidoflavus* NBC_01270 (Jørgensen et al. 2024), a phylogenetically closely related strain (99.1% percent DNA sequence identity), for PTA BGC heterologous expression.

To further improve PTA production in the heterologous hosts, we employed a model-guided workflow to rationally design the fermentation process. Genome-scale metabolic models (GEMs) are comprehensive representations of cellular metabolism that integrate gene–protein–reaction associations into a stoichiometric framework (Gu et al. 2019). Using constraint-based approaches such as flux balance analysis (FBA), GEMs enable prediction of metabolic flux distributions under defined conditions and can guide optimization of nutrient composition and metabolic pathways (Fang et al. 2020). By combining GEM-based simulations with experimental validation, it is possible to systematically identify strategies to enhance production of target metabolites while reducing reliance on empirical trial-and-error (Simeonidis and Price 2015; Yamada et al. 2026).

Together, we achieved over a 20-fold increase in PTA titers in heterologous hosts compared to the reported native producer and established the foundation for further improvement and scale-up of PTA biosynthesis.

## Material and methods

### Bacterial strains, plasmids and reagents

Strains and plasmids used in this study are listed in Table S1. One Shot™ Mach1™ T1 Phage-Resistant chemically competent *E. coli* (ThermoFisher Scientific Inc., USA) was used for CRISPR-Cas9 plasmids construction and maintenance. *E. coli* strain NEB10β (New England Biolabs, MA, USA) harboring pBE14 (Addgene) was used for CAPTURE cloning experiments. *E. coli* ET12567 (pUZ8002) was used as donor strain in conjugation experiments with *Streptomyces* heterologous hosts. *S. albidoflavus* J1074 and NBC_01270 were used for heterologous expression of PTA BGC cloned from *S. iranensis* HM 35. *S. albidoflavus* NBC_01270 was obtained from the NBC strain collection (Jørgensen et al. 2024) (DTU Biosustain). *S. iranensis* HM 35 was obtained from DSMZ. Q5 High-Fidelity 2X Master Mix was used for all PCR reactions following the manufacturer’s instructions. Restriction enzymes, EnGen Lba Cas12a (Cpf1), NEBuffers, T4 DNA polymerase, *E. coli* DNA ligase, dNTPs, NAD^+^ and Q5 High-Fidelity 2X Master Mix were purchased from New England Biolabs (Ipswich, MA, USA). crRNAs and DNA oligonucleotides were ordered from Integrated DNA Technologies (Coralville, IA, USA) and are listed in Table S2.

### Bacterial culture conditions

*E. coli* strains were grown in liquid or solid LB medium (5.0 g/L yeast extract, 10.0 g/L peptone, 10.0 g/L NaCl, 15 g/L agar) at 37 °C. *Streptomyces* strains were grown on SFM agar medium (20.0 g/L mannitol, 20.0 g/L soya flour, 20.0 g/L agar, 10 mM MgCl_2_) at 30 °C. *Streptomyces* spores suspension were prepared by collecting spores from 5 to 7 days SFM agar plates in 6 mL 2xYT media and used for interspecies conjugation or to inoculate precultures. For genomic DNA isolation, *S. iranensis* was cultivated in 40 mL ISP2 medium using 250 mL baffled flasks at 30 °C, 220 rpm for 5–7 days. For preculture, spore suspension was inoculated in ISP2 or Tryptic Soy Broth (BD Difco™) medium. Fermentation media for flasks, TOM, Chi.Bio or ambr® 250 cultivation were: minimal medium (MM) [6 g/L (NH_4_)_2_SO_4_, 1.86 g/L citric acid, 0.21 g/L FeCl_3_, 3.33 mL/L 2 M MgSO_4_, 0.1 M phosphate buffer pH 7.0, 1.6 mL/L trace metal solution (20.4 g/L H_2_SO_4_ 96%, 50 g/L sodium citrate tribasic dihydrate, 16.75 g/L ZnSO_4_·7H_2_O, 2.5 g/L CuSO_4_·5H_2_O, 1.5 g/L MnCl_2_·4H_2_O, 2 g/L H_3_BO_3_, 2 g/L NaMoO_4_·2H_2_O) and carbon source [14.5 g/L sodium glutamate; 5 g/L mannitol; or 5 g/L mannitol and 0.5 g/L glycerol]; Am2ab (5.0 g/L starch, 20.0 g/L glucose, 2.0 g/L bacteriology peptone, 2.0 g/L yeast extract, 5.0 g/L soya flour, 0.5 g/L KH_2_PO_4_, 0.5 g/L MgSO_4_·7H_2_O, 4.0 g/L NaCl, 2.0 g/L CaCO_3_), ISP2 (4 g/L yeast extract, 10 g/L malt extract, 4 g/L glucose), DNPM (40 g/L dextrin, 7.5 g/L peptone from meat, 5 g/L yeast extract, 21 g/L MOPS) and MS liquid (20 g/L mannitol, 20 g/L soy flour). Unless otherwise specified, all the strains were cultivated in 40 mL medium, 250 mL Erlenmeyer baffled flask, 220 rpm, at 30 °C and analyzed at the end of the 7 days of fermentation. Appropriate antibiotics were supplemented with the following working concentrations: apramycin (50 µg mL^–1^), chloramphenicol (25 µg mL^–1^), kanamycin (50 µg mL^–1^) and hygromycin (100 µg mL ^–1^).

### Biosynthetic pathway cloning

PTA biosynthetic gene cluster was cloned using CAPTURE (Enghiad et al. 2021), with some modification from the original protocol. For Cas12a genomic DNA digestion, two downstream and one upstream crRNAs were selected using CRISPOR (http://crispor.gi.ucsc.edu/) (Concordet and Haeussler 2018) and ordered as 2 nmol Alt-R™ L.b. Cas12a crRNA from IDT Technologies. 2100 ng of each Alt-R™ L.b. Cas12a crRNA (crRNA PTA-up_1 and crRNA PTA-dw_1; or crRNA PTA-up_1 and crRNA PTA-dw_2) were pre-incubated with 5 pmol of EnGen Lba Cas12a (Cpf1), 30 µL of 10 × NEBuffer 2.1 and RNAse-free water at 37 °C for 30 min. 15 µg of genomic DNA was added, mixed by gentle inversion, and incubated overnight at 37 °C, followed by enzyme inactivation at 65 °C for 20 min. Further steps were followed as described previously (Enghiad et al. 2021). DNA was resuspended in 20 µL of 10 mM Tris–HCl pH 8.0, at 4 °C, overnight.

The assembly of linear DNA fragments by the T4 DNA polymerase exo + fill-in method was performed the day after the digestion, followed by transformation in homemade electrocompetent *E. coli* NEB10β (pBE14), as described in the previously (Enghiad et al. 2021). As the cloned region was around 68 kb, pBE48 plasmid was selected together with pBE45 plasmid to be used as templates for PCR amplification of DNA receivers. White colonies obtained from transformation plate containing apramycin were screened by colony PCR (cPCR) with primers binding upstream (STR_PR217 and STR_PR218), center (STR_PR219 and STR_PR220) and downstream (ZHIYAN163 and ZHIYAN164) regions of the target BGC. Clones confirmed by cPCR were isolated using NucleoBond Xtra BAC kit for large construct plasmid DNA (MACHEREY–NAGEL, Germany) and verified by sequencing using Oxford Nanopore R9.4.1 flow cell as described by Alvarez-Arevalo et al. (2023) and base called using Guppy (software version 6.3.8 + d9e0f64, client–server API version 13.0.0). The reads were mapped to reference plasmid using Geneious Prime® 2024.0.7 (minimap2).

### Interspecies conjugations

Transfer of the CRISPR-Cas9 plasmids or the cloned PTA BGC into *Streptomyces* heterologous hosts strains were performed through interspecies conjugations using *S. albidoflavus* J1074 and NBC_01270. *E. coli* ET12567 (pUZ8002) room temperature electrocompetent cells (Tu et al. 2016) were transformed with DNA plasmids and plated on LB plates supplemented with 50 µg/mL apramycin, 25 µg/mL chloramphenicol, and 50 µg/mL kanamycin. Isolated colonies were inoculated overnight in 10 mL LB supplemented with antibiotics. Overnight cultures were harvested and washed twice with 5 mL of fresh LB medium at 4000 rpm. 500 µL of resuspended *E. coli* ET12567 (pUZ8002) cells harboring DNA plasmid were mixed with 500 µL of *Streptomyces* spore suspension and spread on SFM supplement with 10 mM MgCl_2_ plates. Overlays were performed 14–16 h later with 1 mL of ddH_2_O, containing 5 µL of 25 mg/mL nalidixic acid and 15 µL of 50 mg/mL apramycin or 30 µL of 100 mg/mL hygromycin. Exconjugants were picked using toothpicks and transferred to selective SFM plates supplemented with nalidixic acid and apramycin or hygromycin.

### Construction of promoter knock-in plasmid

pCRISPR-Cas9 (Tong et al. 2015) plasmid was modified by replacing the apramycin resistance gene with hygromycin to obtain pCas9h. Linearized pCRISPR-Cas9 (~ 9.7 kb, fragment 1) was obtained by *Apa*I and *BstB*I double digestion followed by gel-purification using NucleoSpin Gel and PCR Clean-up (Macherey–Nagel) kit according to the suppliers’ instructions. The hygromycin cassette (fragment 2; 1223 bp) was PCR amplified from pKC1218(Bierman et al. 1992), with primers STR_PR489 and STR_PR490 containing 20 bp homology to linearized pCRISPR-Cas9 and 20 bp homology to fragment 3. Fragment 3 (663 bp) was PCR amplified from pCRISPR-Cas9 backbone with primer STR_PR485 and STR_PR486. All three fragments were assembled using NEBuilder HiFi DNA Assembly (New England BioLabs Inc., USA), using 70 ng of linearized vector, twofold excess of each building block and incubation at 50 °C, 1 h. The whole plasmid sequencing was performed by Plasmidsaurus using Oxford Nanopore Technology with custom analysis and annotation.

A helper plasmid was subsequently obtained to facilitate promoter knock-in as described by Zhang M. et al. (Zhang et al. 2017). A 178 bp insert was designed to include adapters 1 and 2, *kasO*p* promoter(Wang et al. 2013) and 25 bp homology arms specific to the pCas9h plasmid. The insert was ordered and synthesized as a gBlock gene fragment by Integrated DNA Technologies (IDT). The *kasO*p*-gBlock was cloned into *Hind*III and *Eco*RI linearized pCas9h using NEBuilder HiFi DNA Assembly to obtain pCas9h-*kasO*p*.

The genome editing plasmid (pCas9h-*kasO*p*-ptaA) was obtained by inserting repair templates into pCas9h-*kasO*p* using NEBuilder HiFi DNA Assembly. A downstream repair template (~ 1 kb) was inserted into *Spe*I linearized pCas9h-*kasO*p* and the obtained construct was linearized by double digestion with *BamH*I and *Xba*I to be assembled with upstream repair template (1 kb). Finally, sgRNAs were designed and selected using CRISPy-web (Blin et al. 2016), then inserted into *Nco*I restriction site of pCas9h-*kasO*p*-ptaA, as previously described (Tong et al. 2015). Repair template and sgRNA insertion was validated by plasmid Sanger sequencing (Mix2Seq kit – Eurofins Genomics, Germany) using primers nCas9-rev/fw and SDT_PR22.

All assembly reactions were transformed in *E. coli* Mach1™ T1^PR^ competent cells and plasmids selected on LB agar supplemented with hygromycin.

### Promoter engineering in *Streptomyces* heterologous host

pCas9h-*kasO*p*-*ptaA* was transferred to J1074-PTA or NBC1270-PTA by interspecies conjugation. Strains harboring the plasmid were pre-screened by colony PCR with primers STR_PR294 (binding outside repair template) and STR_PR1035. For plasmid curation, positive clones were cultivated in non-selective ISP2 liquid medium at 40 °C, overnight, and plated on non-selective SFM plates. Isolated colonies were picked separately on selective (plates containing hygromycin) and non-selective SFM plates for identification of plasmid-free clones. Hygromycin-sensitive clones were then subjected to final validation of promoter knock-in by cPCR and PCR product Sanger sequencing (Mix2Seq Kit, Eurofins Genomics, GE) with primer STR_PR1035.

### Genome sequencing and analysis

Exconjugants from PTA BGC integration experiments were screened by colony PCR and genomic DNA were isolated from *Streptomyces* plates using DNeasy PowerLyzer PowerSoil Kit (Qiagen, Germany). For efficient cells lysis, bead beating was performed using a TissueLyser LT (Qiagen, Germany) for 10 min. Genomic DNA purity was verified using NanoDrop™ 2000 and concentration was measured using Qubit 4 Fluorometer (Thermo Fisher Scientific Inc., USA) for concentration measurements. DNA fragmentation was attested by gel electrophoresis (0.8% agarose gel). The samples were sequenced on an Oxford Nanopore R9.4.1 flow cell as described by Alvarez-Arevalo et al. (2023) and basecalled using Guppy (software version 6.3.8 + d9e0f64, client–server API version 13.0.0). Geneious Prime® 2024.0.7 was used to map reads to the reference genome and for mappings visualization.

### Fed-batch fermentation

Fed-batch fermentations were performed in an ambr® 250 fermentation system (Sartorius, Germany) using strains J1074-PTA-*kasO*p* and NBC1270-PTA-*kasO*p*. The bioreactors were inoculated to an OD600 nm of 0.2 and the fermentation started in batch phase with 100 mL minimal media containing 5 g/L mannitol or 5 g/L mannitol + 0.5 g/L glycerol as carbon sources. Batch fermentations were performed until complete C-source consumption, marked by a 50% decline in the CO_2_ formation rate, triggering an automatic switch to an exponential fed-batch operation with continuous supply of the feed media (200 g/L mannitol or 200 g/L mannitol supplemented with 20 g/L glycerol). Initial feed rate (F₀) was 0.16 mL h-1 and increased exponentially over time to a set specific growth rate (µ_set_) of 0.11 h^−1^. Cultivations in the ambr® 250 system were performed at 30 °C. Dissolved oxygen was maintained at 40% through cascade PID control, adjusting stirring speed (1000–4000 rpm), airflow (0.1–0.25 L min^−1^), and oxygen enrichment (0–20%). The pH was kept constant at 7.0 by automatic addition of 8% (v/v) ammonium hydroxide and 4.8 M phosphoric acid. Samples were taken for LC–MS analysis at 24, 48, 72 and 96 h for PTA production analysis. Carbon evolution rate (CER) growth (mmol/L/h) was the main parameter triggered during the fermentation to control the process from which µ_max_ were calculated (Tables S5, and S6).

### Genome-scale metabolic model refinement and validation

*Salb*-GEM was used as the base model (Kittikunapong et al. 2021). Phenomics data were used to validate the performance of the GEMs (Jönsson et al. 2025). Activity index values were obtained through the phenome module of Ductape (Galardini et al. 2014). An activity index threshold of 3 was selected to compare growth between model simulation and experimental data. For gap-filling on substrates that were false negatives, a custom pipeline is utilized. In this pipeline, reactions from *Sco*GEM (Sulheim et al. 2020) (first), and reactions from the BiGG Models (Norsigian et al. 2019) database (second) were adopted. Then, gene-protein-reaction (GPR) associations were established where possible for *S. albidoflavus* genome. Finally, manual curation was performed to resolve any remaining inconsistencies, remove redundancies, and confirm overall coherence. This process is documented in GitHub repository (https://github.com/biosustain/salb_GEM_biosustain).

The PTA production biosynthetic pathway was incorporated as an overall reaction. To construct the reactions in the biosynthetic pathway, MIBiG (Zdouc et al. 2025) was used to determine the intermediate steps. Where necessary, compound analysis was performed using MolView (https://molview.org) for structural identification and nomenclature, ChemSpider (https://www.chemspider.com) for physicochemical property determination, and available literature to identify established biosynthetic reactions (Karp et al. 2019; Blin et al. 2023).

### Metabolites identification and quantification

PTA BGC metabolites, including PTA and elaiophylin, were identified and quantified using the methods previously described (Yang et al. 2023). Fermentation cultures were processed by mixing 1-mL supernatant with 1 mL of ethyl acetate, sonicated for 30 min and centrifuged at > 12,000 × g for 10 min. 800 µL supernatant was transferred to a new tube and samples dried under vacuum, followed by resuspension with 500 µL methanol. Ultra-high-performance liquid chromatography-diode array detection-quadrupole time-of-flight mass spectrometry (UHPLC-DAD-QTOFMS) was performed on an Agilent Infinity 1290 UHPLC system equipped with a diode array detector. Separation was achieved on a Poroshell 120 phenyl-hexyl column (250 × 2.1 mm i.d., 2.7 μm, Agilent Technologies, USA) held at 60 °C. A 5-μL injection of each sample was eluted at a flow rate of 0.35 mL min^−1^ using a linear gradient from 10% acetonitrile in Milli-Q water buffered with 20 mM formic acid increasing to 100% over 14 min. Each starting condition was held for 3 min before the next run. Mass spectrometry detection was performed on an Agilent 6545 QTOF MS equipped with Agilent Dual Jet Stream electrospray ion source (ESI) with a drying gas temperature of 160 °C, a gas flow of 13 L min^−1^, sheath gas temperature of 300 °C, and flow of 16 L min^−1^. The capillary voltage was set to 4000 V and the nozzle voltage to 500 V in positive mode. MS spectra were recorded as centroid data over an m/z range of 100–1700, and auto MS/HRMS fragmentation was performed at three collision energies (10, 20, and 40 eV), on the three most intense precursor peaks per cycle. The acquisition rate was 10 spectra s^−1^. Data was analyzed using Agilent MassHunter Qualitative Analysis software (Agilent Technologies, USA). Compound identification was performed by molecular weight, confirmed by MS2 fragmentation, and retention time comparison with pteridic acids F/H, as described in previous study (Yang et al. 2023). Quantification was performed using an external calibration curve prepared from serial dilutions of purified pteridic acids F/H as standard compounds. EIC or UV peak areas obtained from HRLC-MS analysis were plotted against known concentrations, and the resulting linear regression equation was applied to calculate compound concentration in the samples. A dilution factor of 0.625 was considered in the formula.

## Results

### Biosynthetic pathway direct cloning and engineering

In this study, we aimed to clone and heterologously express the identified type I PKS biosynthetic gene cluster (PTA BGC) responsible for the biosynthesis of PTA-H, PTA-F, and elaiophylin in *S. iranensis* HM 35 (Fig. 1). The target region comprising 26 genes (68,286 bp) (Fig. 1a) was assembled into the pBE45-pBE48 vector backbone.

To increase cloning efficiency, one crRNA targeting the upstream region and two distinct crRNAs targeting the downstream region of the PTA BGC were designed, resulting in two independent cloning experiments (named “UP1 + DW1-clone1” and “UP1 + DW2-clone2”). White colonies obtained from both cloning approaches were screened by colony PCR. For clone 1, 7 out of 10 screened colonies were positive, whereas all 10 screened colonies were positive for clone 2 (Fig. S1a).

**Fig. 2:**
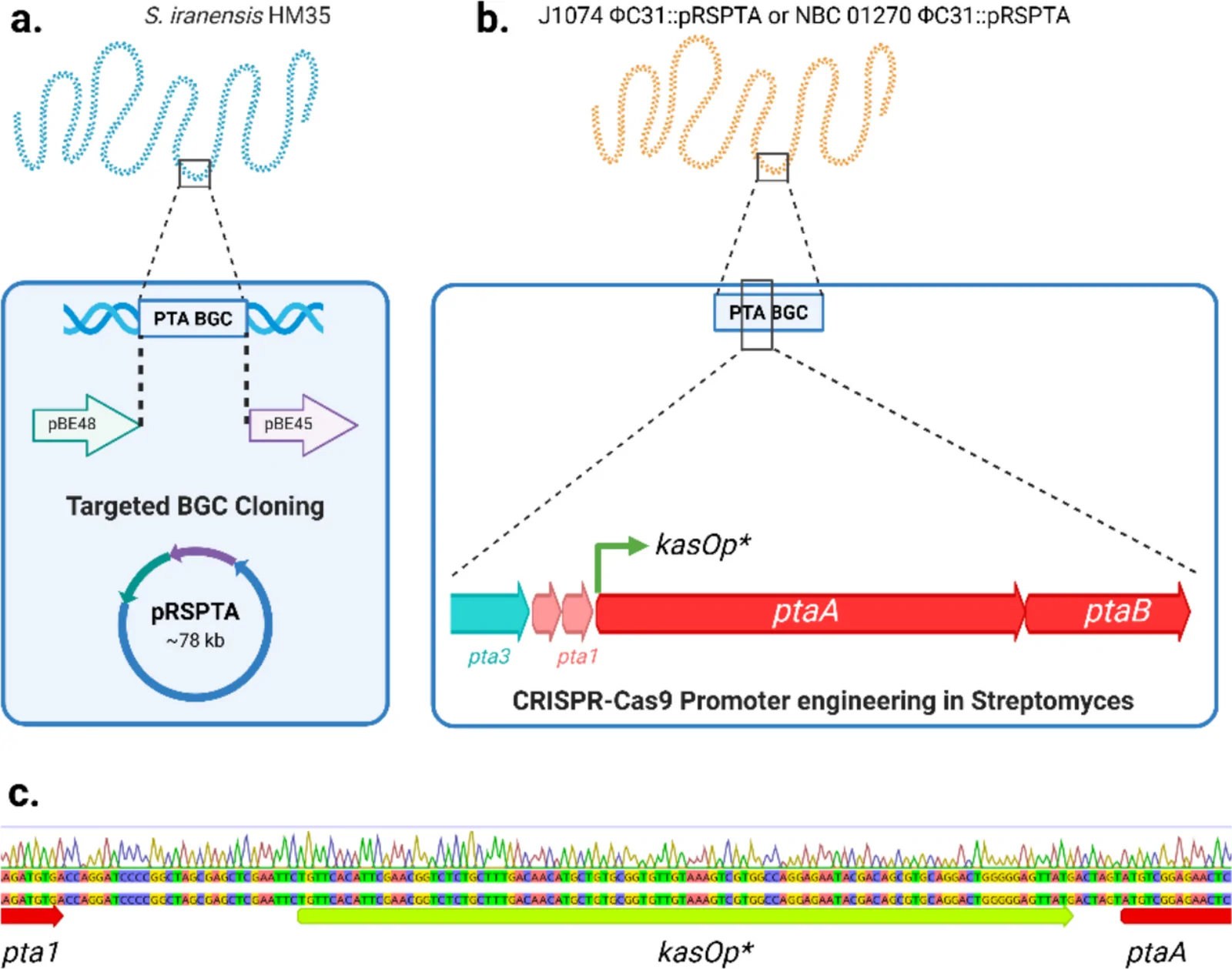
Biosynthetic pathway cloning and engineering. **a **Targeted BGC cloning using CAPTURE system to obtain pRSPTA plasmid (~ 78 kb). **b **Promoter engineering in *Streptomyces* NBC1270-PTA and J1074-PTA using CRISPR-Cas9 system. The constitutive promoter kasOp* was inserted upstream of the *ptaA* gene. **c **Sanger sequencing confirming *kasO*p* knock-in in *Streptomyces* using CRISPR-Cas9 system. *Created in BioRender. Sigrist, R. (2026) *https://BioRender.com/mnnv4cm

One positive colony of each clone was selected for large plasmid extraction and clone 2 was confirmed by Oxford Nanopore sequencing to contain the intact PTA BGC. The obtained BAC was named pRSPTA (~ 78 kb) and refers to the captured PTA BGC into pBE45-pBE48 plasmid (Fig. 2a and Fig. S1b). The cloned PTA BGC was integrated into the heterologous hosts chromosomes at the *attP*-site by interspecies conjugation between the donor strain *E. coli* ET12567 (pUZ8002) harbouring pRSPTA plasmid and *S. albidoflavus* J1074 or *S. albidoflavus* NBC 01270, as heterologous hosts. The exconjugants harbouring the PTA BGC were selected by apramycin resistance phenotype and one clone for each heterologous strain was selected and the correct integration of the BGC was confirmed by Oxford Nanopore sequencing. Correct clones were named NBC1270-PTA and J1074-PTA.

The *Streptomyces* strains containing integrated PTA BGC were further engineered by *kasO*p* knock-in upstream the core biosynthetic gene, *ptaA* (Fig. 2b), using CRISPR-Cas9 system pCRISPR-Cas9 (Tong et al. 2015) plasmid was selected for promoter engineering experiments and modified by replacing apramycin with hygromycin resistance gene to obtain pCas9h. The modified plasmid was used as a template to obtain pCas9h-*kasO*p*-ptaA containing *kasO*p* promoter flanked by ~ 1 kb homology arms to specifically target the region upstream the core biosynthetic genes. The plasmid was transferred to the *Streptomyces*-PTA strains by conjugation. After conjugation and plasmid curation, isolated colonies were screened by colony PCR and PCR products were sequenced by Sanger sequencing to confirm the promoter insertion (Fig. 2c). Correct clones were named NBC1270-PTA-*kasO*p* and J1074-PTA-*kasO*p*.

### Comparative growth analysis of native and heterologous strains

To obtain a better overview of all the strains, *S. iranensis* HM 35 (native), *S. albidoflavus* NBC_01270 and *S. albidoflavus* J1074, a comparative growth curve was obtained using data collect from Chi.Bio system in TSB media and minimal media. Under TSB media condition, the native strain reached the exponential phase after 40 h, while the heterologous strains after 12 h (Figure S2). When cultivated in defined minimal media, the heterologous strain *S. albidoflavus* J1074 performed similarly to TSB condition, achieving exponential phase after 12 h, whereas the native strain showed no growth (Fig. S3). No data was collected for *S. albidoflavus* NBC_01270 in minimal media.

### PTA heterologous expression

Following conjugation and chromosomal integration of the PTA BGC, the resulting strains NBC1270-PTA and J1074-PTA were cultivated in five different media (Am2ab, DNPM, MS liquid, ISP2, and MM) to evaluate production of PTA BGC–related metabolites (Table S3).

No PTA was detected in ethyl acetate extracts from either strain under the tested conditions. However, elaiophylin was detected below the limit of quantification in Am2ab medium for NBC1270-PTA (data not available for J1074) and in MS liquid medium for both strains.

To enhance pathway expression, the constitutive promoter *kasO*p* was inserted upstream of the core biosynthetic gene. NBC1270-PTA-*kasO*p* and J1074-PTA-*kasO*p* were cultivated in ISP2, MM, DNPM, and Am2ab media (Table S3), and culture extracts were analyzed by LC–MS. Elaiophylin was detected in all tested media except ISP2 for both strains. PTA were detected in DNPM and Am2ab for both engineered strains. However, compound concentrations remained below the limit of quantification, preventing accurate titer determination.

### In silico optimization of the PTA biosynthetic pathway through media supplementation

**Fig. 3:**
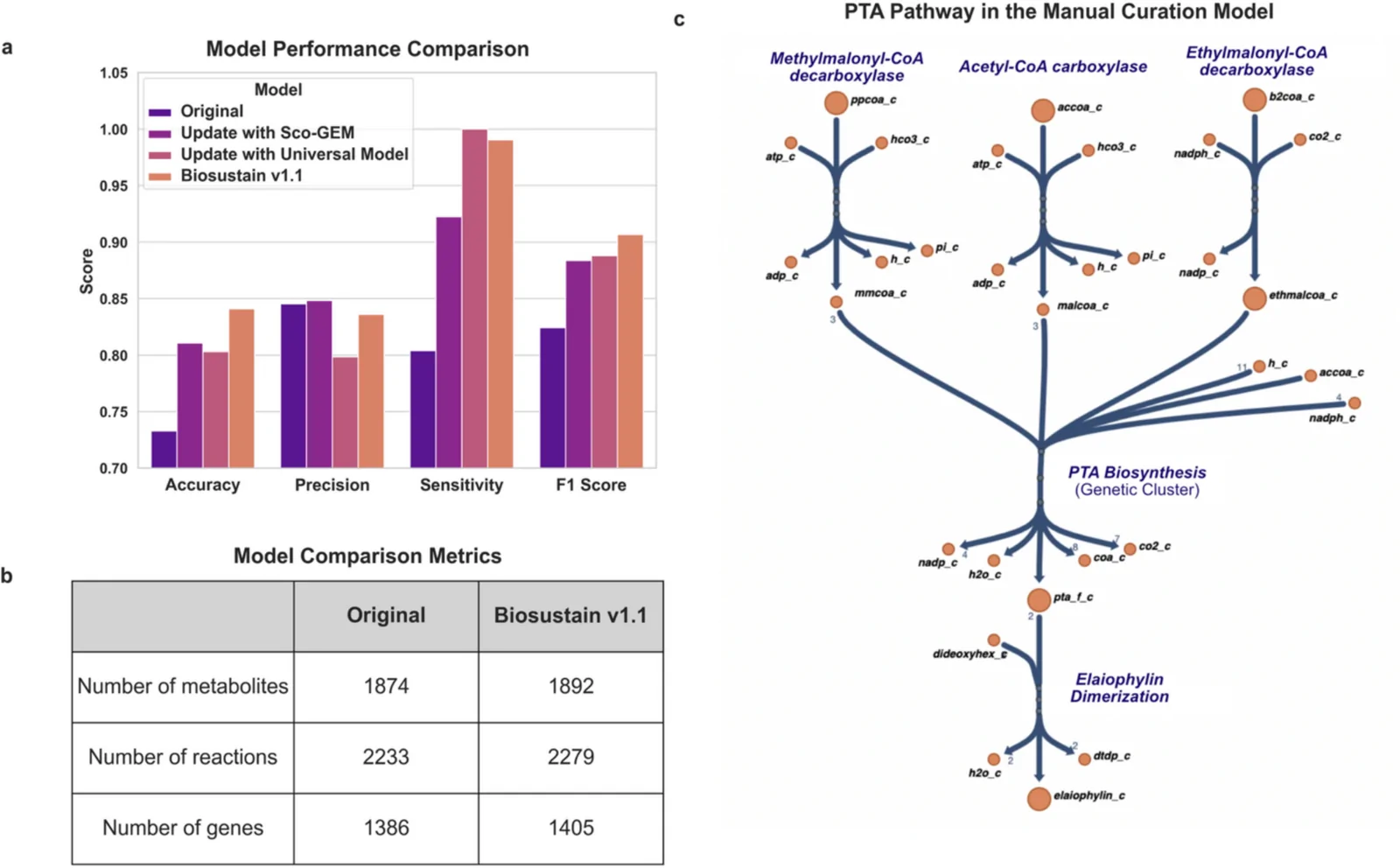
Iterative refinement of the genome-scale model and integration of the PTA pathway. **a **Comparison of model predictive performance evaluated against phenomics data after each refinement step (Original *Salb*-GEM v1.0.1, gap-filling with *Sco*-GEM, gap-filling with Universal Model from BiGG database, and the final Manually Curation Model (*Salb-GEM-Biosustain v1.1*)). Performance is measured by accuracy, precision, sensitivity, and F1 score. **b **Increase in model complexity shown by comparing the number of metabolites, reactions, and genes between the original *Salb*-GEM and the final manually curated model. **c** Representation of the integrated PTA biosynthetic pathway within the manually curated model structure, illustrating precursor pathways feeding into an overall reaction node representing the core *PTA Biosynthesis* steps

**Fig. 4:**
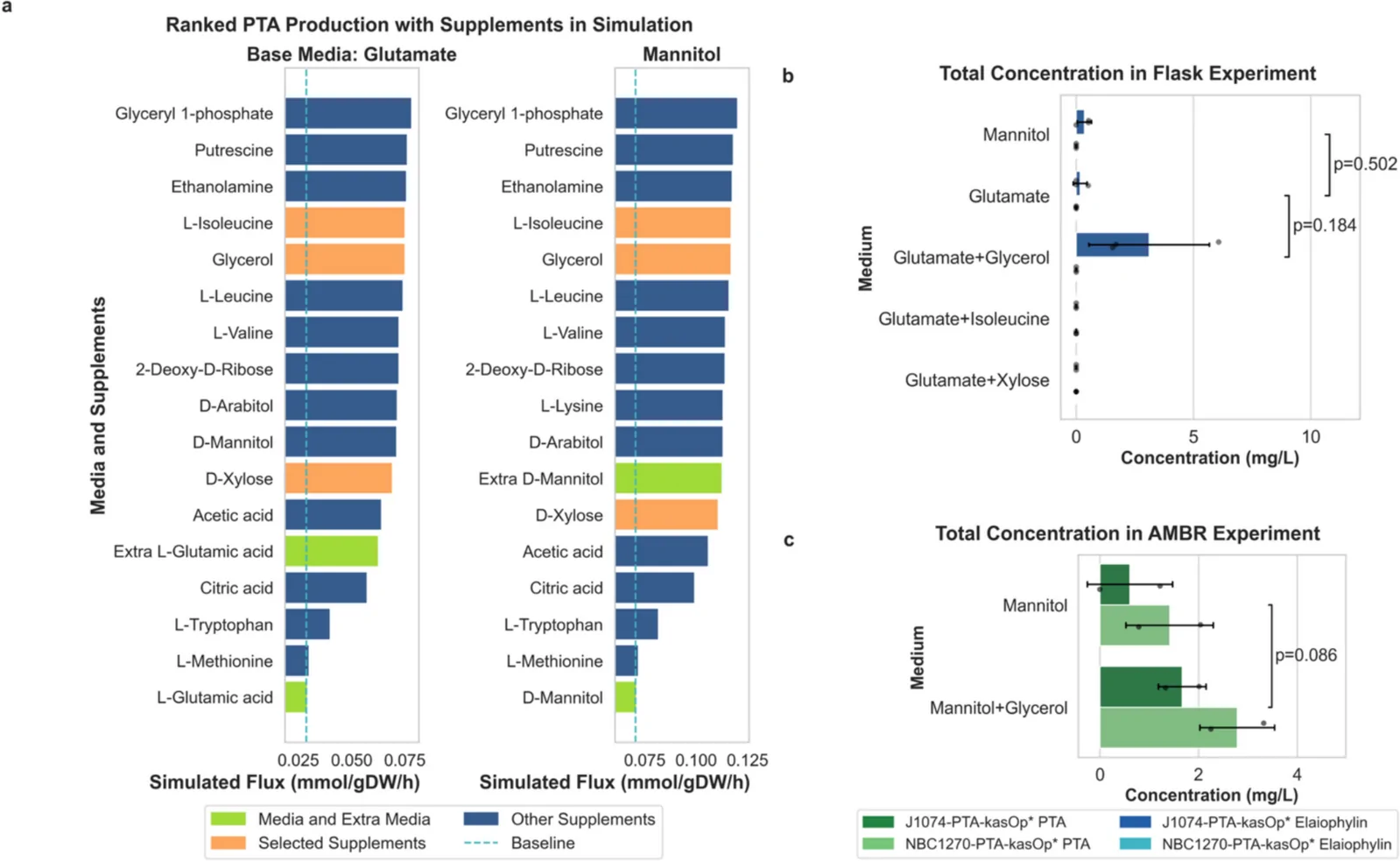
Comparison of genome-scale model predictions and experimental validation for enhancing PTA production through media supplementation. **a **Genome-scale model predictions ranking the effect of individual supplements on PTA production when added to either glutamate or mannitol-based media. Predicted PTA concentrations are compared against the baseline production level (dashed vertical line) achieved with the base media alone. Other supplement candidates can be found in Supplementary Fig. S4. **b **Experimental validation showing total concentration (mg/L) achieved in shake flask cultivation and selected media conditions. Data shown is for J1074-PTA-*kasO*p* (dark blue/green) and NBC1270-PTA-*kasO*p* (light blue/green). PTA (green) were not detected in flask condition. **c **Experimental validation in ambr® 250 bioreactor. Total concentration (mg/L) achieved in mannitol-based medium and mannitol-based medium supplemented with glycerol. Data shown is for J1074-PTA-*kasO*p* (dark blue/green) and NBC1270-PTA-*kasO*p* (light blue/green). Bars in (**b**) and (**c**) represent mean values, overlaid with individual data points representing independent culture replicates. Error bars indicate standard deviation (SD). Statistical significance between selected conditions was determined using an unpaired, two-tailed Welch’s *t*-test to account for unequal variances (*p*-values indicated on plot). PTA concentration: green; elaiophylin concentration: blue

To simulate PTA production, we integrated the biosynthetic pathway into the manually curated model *Salb-GEM-Biosustain v1.1* (Supplementary Discussion; Fig. 3a and b) based on reactions from the PTA pathway (Fig. 3c) through an overall reaction. We conducted simulations with defined media (48 C-mmol/gDW/h equivalent) to evaluate flux compatibility in the engineered model. With the engineered biosynthetic pathway, we made the *Salb-GEM-Biosustain v1.1* model that was used in the predictions. Using the refined, validated, and pathway-integrated *Salb-GEM-Biosustain v1.1* for *S. albidoflavus* J1074, we predicted media supplements that could enhance PTA production by identifying key nutrients and cofactors to optimize the fermentation process. A simulation adding different carbon sources to glutamate- and mannitol-based media was performed, keeping the biomass rate constant while increasing carbon input by 20% (Fig. 4a and Figure S4). Among the top candidates predicted to enhance PTA production, L-isoleucine and glycerol were selected for experimental validation based on their predicted impact, their involvement in central metabolic pathways linked to precursor supply, and their cost-effectiveness and industrial relevance. L-isoleucine was prioritized as a representative amino acid due to its potential contribution to methylmalonyl-CoA biosynthesis, an important extender unit for PTA production. D-xylose, although not among the top-ranked candidates, was included as a low-cost carbon source to explore its potential effect on central carbon metabolism and process feasibility.

### Experimental validation of model predictions

Based on the in silico simulation results, experimental validation was performed in shake-flask cultivations using defined minimal media containing either glutamate or mannitol as the primary carbon source. Supplementation experiments were conducted in glutamate-based medium with the addition of L-isoleucine, glycerol, or D-xylose. For each condition, three replicate flasks were inoculated from the same seed culture.

PTA production in *Streptomyces* hosts was influenced by carbon–nitrogen balance and the nature of the carbon source under minimal conditions (Fig. 4b). Mannitol-based medium slightly outperformed glutamate-based medium, likely because glutamate provides excess nitrogen that can maintain cells in a primary metabolic state and repress secondary metabolism, whereas mannitol favors a metabolic shift toward biosynthetic gene cluster activation while providing efficient flux toward acetyl-CoA and malonyl-CoA required for polyketide biosynthesis. (van Wezel and McDowall 2011) Supplementation effects further highlighted these trends: glycerol enhanced production, consistent with its role as a relatively non-repressing carbon source that supports central metabolic flux and precursor availability, while D-xylose reduced production, likely due to inefficient utilization and limited flux toward key intermediates. (van Wezel and McDowall 2011; Swiatek et al. 2013) Similarly, L-isoleucine supplementation negatively affected production, which may reflect nitrogen-associated regulatory effects and feedback inhibition that suppress the transition to secondary metabolism. (van Wezel and McDowall 2011) Together, these results emphasize that both nutrient composition and regulatory responses to carbon and nitrogen sources critically determine polyketide production in *Streptomyces*.

Further scale-up validation was performed in ambr® 250 bioreactors using fed-batch cultivation. Based on shake-flask results, mannitol-based medium was selected and compared to mannitol-based medium supplemented with glycerol. To establish feeding parameters, a Kuhner TOM shaker system was used to monitor online carbon transfer rate (CTR, mmol L⁻^1^ h⁻^1^) and obtain biomass yield on substrate (Ysx) and maximum specific growth rate (μ_max_, h^−1^) for *S. albidoflavus* J1074 and *S. albidoflavus* NBC_01270.

The initial feed rate (F₀) was calculated according to: *F*_*o*_ = *((µ*_*set*_*)/Y*_*sx*_*)⋅((X*_*o*_*⋅V*_*o*_*)/S*_*feed*_*)*, where Xo is the initial biomass concentration, Vo the initial culture volume, S_feed_ the substrate concentration in the feed, and μ_set_ the specific growth rate set point during the feed phase. The value of μ_set_ was defined as 80% of the average μ_max_ determined for both strains (Table S4). The feed rate at any time point of the fed-batch process was calculated using*: F(t)* = *F*_*o*_*. e*µ_set_⋅t, where *t* represents the time since the feeding onset.

After four days of fed-batch fermentation, PTA were detected under both media conditions. Glycerol supplementation resulted in higher PTA production, reaching 1.7 +/− 0.48 mg/L in J1074-PTA-*kasO*p* and 2.8 +/− 0.76 mg/L in NBC1270-PTA-*kasO*p* (*p* = 0.086) (Fig. 4c).

## Discussion

In this study, we achieved heterologous expression of the type I PKS biosynthetic gene cluster (68 kb) responsible to produce pteridic acids (PTA-F and PTA-H) and elaiophylin through a combined strategy of precise BGC cloning, promoter engineering, and genome-scale model–guided media optimization. To our knowledge, this is the first successful report of heterologous expression of BGC related to PTA-F, PTA-H and elaiophyllin biosynthesis, overcoming previous unsuccessful attempts (Yang et al. 2022), likely due to challenges associated with large PKS assembly lines or insufficient transcriptional activation. The use of the CAPTURE system enabled accurate cloning of the intact 26-genes region into a BAC backbone (pBE45 + pBE48), preserving cluster integrity and regulatory context. *S. albidoflavus* J1074 and the phylogenetically closely related *S. albidoflavus* NBC_01270 (Jørgensen et al. 2024) were selected as heterologous hosts, both sharing 99.1% percent DNA sequence identity (Blastn). Successful chromosomal integration into the two strains established a stable production platform and confirmed that the PTA BGC is functionally transferable across species.

Additionally, the comparison of growth profiles between the heterologous hosts and the native producer revealed clear physiological advantages of the heterologous host strains. Both *S. albidoflavus* strains exhibited rapid growth in complex and defined minimal medium, whereas the native *S. iranensis* HM 35 strain showed long lag phase in rich media and no detectable growth under minimal medium conditions. This also highlights the robustness of the heterologous strain, as defined media provide controlled nutrient conditions that enable small-scale metabolic and fermentation performance to translate more predictably to larger-scale processes (Wentzel et al. 2012).

Initial heterologous expression resulted in detectable production of elaiophylin but not PTA. Because both metabolites arise from the same PKS assembly line and diverge at the level of thioesterase-mediated chain release and dimerization (Fig. 1b), these results suggest that elaiophylin formation is favored over PTA production under basal expression conditions. Following insertion of the strong constitutive promoter *kasO*p* (Wang et al. 2013) upstream of the core biosynthetic gene *ptaA*, both PTA and elaiophylin became detectable, suggesting that gene expression level was a limiting factor in PTA biosynthesis in heterologous hosts.

Proteomics analysis confirmed detection of PTA BGC-associated proteins, including PtaA, PtaB, and PtaC, under cultivation conditions where compound production was observed (Supplementary Methods and Fig. S5). Because native and engineered strains were analyzed under their respective productive media conditions, these data provide qualitative support for pathway expression at the protein level rather than a direct quantitative comparison between strains. Despite this, titers remained low and below the limit of quantification, highlighting that promoter engineering alone is insufficient to fully optimize production. Additional constraints such as enzyme efficiency, precursor supply, or competing catalytic activities within the thioesterase domain likely restrict flux distribution toward PTA formation.

To further enhance PTA production, we adopted genome-scale metabolic modelling. First, we refined and validated the previously published *S. albidoflavus* J1074 model (i.e. Salb-GEM). Iterative gap filling and manual curation improved predictive accuracy against phenomics data from 73% to 84%, increasing confidence in subsequent simulations and yielding the updated model, *Salb-GEM-biosustain v1.1*. Second, we incorporated the PTA pathway as an overall reaction to enable evaluation of precursor supply under defined constraints. During simulations, biomass generation was maintained as the primary objective; however, by fixing the growth rate to experimentally observed values, we could selectively maximize metabolic flux through the PTA pathway. To evaluate optimal carbon sources, we used a mannitol-based medium as our baseline in silico medium, as our initial experiments demonstrated it yielded the highest growth rate in batch fermentation. We compared this against L-glutamate-based medium, since Wentzel et al. (2012) previously established it as a superior carbon source to glucose for the closely related *S. coelicolor* A3(2). This allowed us to identify media conditions that maximize precursor availability, assuming enzyme capacity is not the primary bottleneck. Lastly, in silico supplementation analysis identified L-isoleucine, glycerol, and D-xylose as candidate nutrients predicted to increase flux toward PTA synthesis. L-isoleucine is a precursor for propionyl-CoA biosynthesis, while glycerol and D-xylose are abundant, low-cost alternative carbon sources capable of enhancing metabolic flux toward PTA (Orts et al. 2008).

Experimental validation showed a strong trend toward increased PTA titers with glycerol supplementation in fed-batch cultivation, whereas L-isoleucine and xylose had no measurable effect. Although the improvement with glycerol did not reach statistical significance (*p* = 0.086), the consistent trend across strains indicates that carbon redistribution can influence PTA biosynthesis. The partial agreement between predictions and experiments highlights limitations of stoichiometric models, which do not capture enzyme kinetics, regulatory effects, or intermediate toxicity—factors particularly relevant for complex polyketide pathways.

Fed-batch fermentation yielded 1.7 mg/L and 2.8 mg/L PTA in J1074-PTA-*kasO*p* and NBC1270-PTA-*kasO*p*, respectively, representing over 20-fold increase compared to the native producer (~ 0.08 mg/L and 0.02 mg/L) under comparable conditions. Industrial production of microbial natural products typically requires titers in the high mg/L to g/L range, whereas initial heterologous expression systems generally yield only mg/L levels (Park et al. 2021). Thus, while the titers achieved here demonstrate successful pathway expression and functionality, further optimization will be necessary to approach industrial relevance. The observed improvement highlights the combined impact of heterologous expression, promoter engineering, and model-guided media optimization. Future efforts may focus on additional pathway engineering, including improving precursor supply, refining enzyme kinetics within the PKS assembly line, or engineering thioesterase specificity to enhance product yield.

Overall, this work establishes a functional heterologous platform for PTA biosynthesis and demonstrates that integration of synthetic biology tools with genome-scale modeling can guide rational improvement of complex natural product pathways. Beyond PTA production, the strategy presented here provides a transferable framework for activating and optimizing large type I PKS clusters in genetically tractable *Streptomyces* hosts. Given the role of PTA compounds as plant growth–promoting agents, these findings contribute not only to natural product biosynthesis research but also to the development of sustainable agricultural biotechnologies.

## Supplementary Information

Below is the link to the electronic supplementary material.ESM 1(PDF 2.61 MB)

## Data Availability

The updated genome scale model is available at https://github.com/biosustain/salb_GEM_biosustain/tree/main/model. The corresponding experimental data, model curation, analysis and figure creation are available at https://github.com/biosustain/salb_GEM_biosustain.
