# Supplementary material for: Heterologous production of a plant biostimulant in Streptomyces albidoflavus
Source: Appl Microbiol Biotechnol. 2026 Jun 20;110:261. doi: 10.1007/s00253-026-13915-w (PMC13529632; doi:10.1007/s00253-026-13915-w)
Supplement: Supplementary file 1 [file 253_2026_13915_MOESM1_ESM.pdf]

Heterologous production of a plant biostimulant in *Streptomyces albidoflavus*

Renata Sigrist[1,4], Te Chen[1,4], Marta Reventós Montané[1,4], Peter Gockel[1,4], Yijun Qiao[2], Mathias Joensson[3,4], Zhijie Yang[2], Tilmann Weber[2,4], Ling Ding[2], Emre Özdemir[1,4*] and Lei Yang[1,4*]

[1] Current: The Novo Nordisk Foundation Biotechnology Research Institute for the Green Transition, Technical University of Denmark, 2800 Kongens Lyngby, Denmark

[2] Department of Biotechnology and Biomedicine, Technical University of Denmark, 2800 Kongens Lyngby, Denmark

[3] Department of Microbiology and Immunology, The Peter Doherty Institute for Infection and Immunity, University of Melbourne, Melbourne, Victoria, Australia

[4] The Novo Nordisk Foundation Center for Biosustainability, Technical University of Denmark, Kgs. Lyngby, 2800, Denmark

*** Corresponding authors:**

Lei Yang – email: leya@dtu.dk

Emre Özdemir – email: emoz@dtu.dk

Supplementary Information

Supplementary Methods

Proteomic samples preparation

To evaluate expression of proteins associated with the PTA BGC, proteomic analyses were performed on selected native and engineered strains under cultivation conditions where PTA or elaiophylin compounds were detected. NBC1270-PTA was analyzed from cultures grown in MS-liquid medium, while engineered strains J1074-PTA-*kasO*p* and NBC1270-PTA-*kasO*p* were analyzed from cultures grown in all selected media (DNPM, ISP2, Am2ab and MM). Cells were harvested after 7 days cultivation and froze in liquid nitrogen.

3-mm zirconium oxide beads were added to each sample followed by addition of 100 µL lysis buffer (6 M guanidinium hydrochloride, 5 mM Tris (2-carboxyethyl) phosphine, 10 mM Chloroacetamide, 100 mM Tris-HCl pH 8.5) pre-heated at 99 °C. Samples were placed in Tissuelyser at 25 Hz for 5 min for further, followed by boiling in heat block at 99 °C for 10 min, 1800 rpm. After centrifugation, supernatant was collected and mixed with 50 µL of 50 mM ammonium bicarbonate. Protein concentration was measured from supernatant using the BCA protein assay kit (Thermo Fisher Scientific, Waltham, MA, USA). The obtained total proteins were digested with 20 µL of 0.1 µg/µL trypsin and Lys-C mixture, by incubation at 37 °C overnight. After the digestion, 10 µL of 10% trifluoroacetic acid (TFA) were added to the peptide solution and mixed vigorously. The mixture was centrifuged at 14,000× *g* for 15 min and desalted using Stage Tip C18 columns. The peptides bound to the Stage Tip were eluted with 0.1% TFA and 80% acetonitrile. After the desalting, the peptides were dried by a centrifugal evaporator and re-dissolved in 0.1% TFA for LC-MS analysis.

LC–MS/MS-based proteomic analysis

Peptides were loaded onto a 2 cm C18 trap column (ThermoFisher 164946), connected in-line to a 15 cm C18 reverse-phase analytical column (Thermo EasySpray ES904) using 100% Buffer A (0.1% Formic acid in water) at 750 bar, using the Thermo EasyLC 1200 HPLC system, and the column oven operating at 30°C. Peptides were eluted over a 70-minute gradient ranging from 10% to 60% of 80% acetonitrile, 0.1% formic acid at 250 nl/min, and the Orbitrap Exploris instrument (Thermo Fisher Scientific) was run in DIA mode with FAIMS ProTM Interface (ThermoFisher Scientific) with CV of -45 V. Full MS spectra were collected at a resolution of 120,000, with an AGC target of 300% or maximum injection time set to ‘auto’ and a scan

range of 400–1000 m/z. The MS2 spectra were obtained in DIA mode in the orbitrap operating at a resolution of 60.000, with an AGC target 1000% or maximum injection time set to 'auto', a normalised HCD collision energy of 32. The isolation window was set to 6 m/z with a 1 m/z overlap and window placement on. Each DIA experiment covered a range of 200 m/z resulting in three DIA experiments (400-600 m/z, 600-800 m/z and 800-1000 m/z). Between the DIA experiments a full MS scan is performed. MS performance was verified for consistency by running complex cell lysate quality control standards, and chromatography was monitored to check for reproducibility.

Proteomics Data Analysis

The raw files were analyzed using SpectronautTM (version 17) spectra were matched against the Uniprot *Streptomyces iranensis* (Taxonomy ID: 576784) or *Streptomyces albus* (Proteome: UP00003152) database together with PTA biosynthetic gene cluster (BGC) sequence. Dynamic modifications were set as Oxidation (M) and Acetyl on protein N-termini. Cysteine carbamidomethyl was set as a static modification. All results were filtered to a 1% FDR, and protein quantitation done on the MS1 level. The data was normalized by RT dependent local regression model (Callister et al. 2006), protein groups were inferred by IDPicker and imputation by background signal.

Supplementary Discussion

Refinement and Validation of the Genome-Scale Model

To further improve the production of PTA, we employed genome-scale metabolic modeling for systemically analyzing strain growth condition, a powerful tool for predicting alternative cultivation strategies and improving flux of targeted metabolic pathways (Hwang et al. 2014).

A metabolic model for *Streptomyces albus* J1074 – *Salb-GEM* – has been published by (Kittikunapong et al. 2021). We evaluated the model performance of *Salb-GEM*, through phenomics data (Acin-Albiac et al. 2020) generated by (Jönsson et al. 2025) using a Biolog system. The data on utilization of 384 different substrates covered various carbon, nitrogen, phosphorus, and sulfur sources. For each substrate, we calculated cellular activity through growth signals. The signal was then converted to an activity index using dphenome package, DuctApe (Galardini et al. 2014). This activity index was used to compare with simulated growth profiles from *Salb-GEM* model. Out of 384 experimentally used substrates from Biolog, 131 metabolites are possible to be tested with the *Salb-GEM*.

The simulation of the original *Salb*-GEM model was able to match 73% of the cases from the tested activity indices. To improve the model, we performed a targeted gap filling. Overall change in model

performance is illustrated by the metrics (accuracy, precision, sensitivity, and F1 score) (**Error! Reference source not found.**a) and the corresponding confusion matrices. Compared with the original *Salb*-GEM model, the final manually curated model (*Salb-GEM-Biosustain v1.1*) added 12 metabolites and 38 reactions (**Error! Reference source not found.**b). With this, *Salb-GEM-Biosustain v1.1* was able to uptake 132 metabolites and 84% of testable substrates that showed correct activity.

Supplementary Tables

Table S1: Strains used in this study.

Strain or plasmid	Description	Source
E. coli NEB10β (pBE14)	*Δ(ara-leu) 7697 araD139 fhuA ΔlacX74 galK16 galE15 e14- Φ80dlacZΔM15 recA1 relA1 endA1 nupG rpsL* (Str^R) *rph spoT1 Δ(mrr-hsdRMS-mcrBC),* carrying pBE14 plasmid for CAPTURE cloning	New England Biolabs, U.S.A
E. coli Mach1™ $T1^{PR}$	*F⁻ φ80lacZΔM15 ΔlacX74 hsdR(*r_K*–, *m_K*+) ΔrecA1398 endA1 tonA, E. coli* for routine cloning	ThermoFisher Scientific Inc., U.S.A
E. coli ET12567 (pUZ8002)	*Escherichia coli* for conjugation, *dam-13::Tn9 dcm-6 hsdM* CmlR, carrying helper plasmid pUZ8002	(MacNeil et al. 1992)
S. albidoflavus J1074	Wild type for heterologous expression of PTA BGC.	(Zaburannyi et al. 2014)
Streptomyces albidoflavus NBC_01270	Wild type for heterologous expression of PTA BGC.	NBC collection (Jørgensen et al. 2024);<br>NCBI access number: GCA_036239155.1
Streptomyces iranensis	Wild type, PTA BGC donor.	Prof. Ling Ding (DTU Bioengineering, Denmark)
J1074-PTA	*S. albidoflavus* J1074 with PTA BGC integrated (J1074 ΦC31::pRSPTA)	This study
J1074-PTA-*kasO*p*	J1074-PTA with *kasO*p* upstream *ptaA*	This study
NBC1270-PTA	*S.* sp. NBC_01270 with PTA BGC integrated (1270 ΦC31::pRSPTA)	This study
NBC1270-PTA-*kasO*p*	NBC1270-PTA with *kasO*p* upstream *ptaA*	This study
pCRISPR-Cas9	Temperature sensitive plasmid, tsr, aac(3)IV, oriT, *to* and *fd* terminator, PtipA, sgRNA scaffold, *cas9*	(Tong et al. 2015)
pCRISPR-Cas9-hyg (pCas9h)	pCRISPR-Cas9 derivative with hyg^R	This study
pCas9h-*kasO*p*	pCas9h with adapters 1 and 2 to facilitate assembly of repair templates upstream and downstream of *kasO*p*	This study

pCas9h-*kasO*p*-*ptaA*	pCas9h-*kasO*p* containing both repair templates for genome editing in *Streptomyces.*	This study
pRSPTA	Captured PTA BGC. *pta*, aac(3)IV, ori2,	This study
pBE14	In vivo Cre-lox circularization in *E. coli*	Addgene (cat. #174653)
pBE45	CAPTURE method receiver plasmid for *Streptomyces* heterologous expression	Addgene (cat. #174655)
pBE48	CAPTURE method BAC origin of replication receiver plasmid	Addgene (cat. #174656)
pKC1218-hyg	Hyg^{R} *lacZa* MCS rep^{SCP2*}	(Bierman et al. 1992) (Prof. S. Zotchev, Uni Vienna)

Table S2. Oligonucleotides and crRNAs for this study.

**Primer Name**	**Sequence**	**Notes**
STR_PR217_fw	CCCGACTATCTCCACCGTGAG	Screening primer binding PTA upstream region
STR_PR218_rv	AACAGGACATTGAGCGAGGTG	Screening primer binding PTA upstream region
STR_PR219_fw	CTGAACTCGATGAAGCCGCTC	Screening primer binding PTA center region
STR_PR220_rv	GATCTGACGGCCTGATGAACC	Screening primer binding PTA center region
ZHIYAN163_fw	TCACTGGTGAGCAGCGATC	Screening primer binding PTA downstream region
ZHIYAN164_rv	GACCGTGTCCAATGAGCTG	Screening primer binding PTA downstream region
STR_PR264	ACATCCGGCGCGTTTCCCACCCAGATCTTTCCGTCCGTTgacgctcagtggaacgaaaac	pBE45 forward receiver primer
STR_PR265	CCGGGCCCCACGTTTGGTCCGGACTCTCACGGGCACTTGGacagcgacacacttgcatcg	pBE48 reverse receiver primer
pBE45-rv	ATCTTTATAGTCCTGTCGGGTTTCG	Universal receiver primer
pBE48-fw	TTACCAATGCTTAATCAGTGAGGCACC	Universal receiver primer
STR_PR485	CCTCGGCCACGACGATGCTG	Building block 2 amplification
STR_PR486	GGGCCGTACGCTGGTACTGC	Building block 2 amplification
STR_PR489	GCAGTACCAGCGTACGGCCCGGTCTGACGCTCAGTGGAAC	Hygromycin cassette amplification (BB1)
STR_PR490	TCGCTTGGTCGGTCATTTCCCGTTCCCGCCAGCTAGAG	Hygromycin cassette amplification (BB1)
STR_PR982	CCGGTTGGTAGGATCGACGGcgactggactgaccatgatcGTTTTAGAGCTAGAAATAGC	gRNA_1 oligonucleotide for CRISPR plasmid
STR_PR983	CCGGTTGGTAGGATCGACGGcatggtcagtccagtcgcgtGTTTTAGAGCTAGAAATAGC	gRNA_2 oligonucleotide for CRISPR plasmid
STR_PR984	CGGCGGCGACTGAAAGCTTTCTAGAAGCTCAGCAAGACGGACTAC	PTAa upstream repair template_fw

STR_PR985	ATTCGAGCTCGCTAGCCGGGGATCCTGGTCACATCTTCTTGAGCG	PTAa upstream repair template_rev
STR_PR987	CAGGACTGGGGGAGTTATGACTAGTATGTCGGAGAACTCCCTGATG	PTAa downstream repair template_fw
STR_PR988	CTGGATACTGACTTTTCACACTAGTCTTGATGAGACCGGTGATTC	PTAa downstream repair template_rev
nCas9_rv	GGAAAGACGACAAAACTTTAGATCTGGG	Screening/sequencing RT
nCas9_fw	GACCCTGATCCACCAGAGCA	Screening/sequencing RT
SDT_PR22		Screening/sequencing gRNA
SDT_PR1035	GAGCTGACCAATCTGGAACTC	*kasO*p* promoter screening
SDT_PR1034	GACATGCCATACCGACGAC	*kasO*p* promoter screening
STR_PR324	TGGCCACGACTTTACAACACC	*kasO*p* promoter screening
STR_PR294	GTTCGGTGTCTCGTGGTTGA	*kasO*p* promoter screening
**crRNA name**	**Sequence**	**PAM**
crRNA PTA-up_1	GTCCGGACTCTCACGGGCACT	TTTG
crRNA PTA-dw_2	CCACCCAGATCTTTCCGTCCG	TTTC
crRNA PTA-dw_1	GTTACGTTATGCCGGAATTAG	TTTG

Table S3. Production of target compounds in heterologous strains cultivated in shake flasks under different media conditions. (NA: data not available; ND: not detected).

	**Strain**			
**Media**	**J1074-PTA**	**NBC1270-PTA**	**J1074-PTA -*kasO*p***	**NBC1270-PTA -*kasO*p***
**Am2ab**	NA	Elaiophylin	Elaiophylin; PTA	Elaiophylin; PTA
**DNPM**	ND	NA	Elaiophylin; PTA	Elaiophylin; PTA
**MS liq**	Elaiophylin	Elaiophylin	NA	NA
**ISP2**	NA	NA	ND	ND
**MM**	ND	ND	Elaiophylin	Elaiophylin; PTA

Table S4. Maximum specific growth rates (µmax) obtained for *S. albidoflavus* J1074 and *S. albidoflavus* NBC_01270 in TOM shake system. Data based on integrated CTR (mmol L^{-1} h^{-1}) measurement.

**Strain**	**System**	**Carbon source**	**µmax (batch phase) (h^{-1})**
***S. albidoflavus* J1074**	TOM	Glutamate	0.0900
***S. albidoflavus* J1074**	TOM	Glucose + Glutamate	0.0838
***S. albidoflavus* J1074**	TOM	Mannitol	0.1305
**NBC_01270**	TOM	Glutamate	0.0780
**NBC_01270**	TOM	Glucose + Glutamate	0.0640
**NBC_01270**	TOM	Mannitol	0.1400

Table S5. Specific growth rates (µmax) measured from CO2 output during the batch growth phase for J1074-PTA-kasOp* in the ambr®250 system. Data based on integrated CTR (mmol L^{-1} h^{-1}) values.

Condition	Replicate	µmax (h^{-1})	µmax (mean, h^{-1})	± SD	CV (%)
Mannitol	R16_1	0.093	0.151	0.058	38.6
Mannitol	R17_2	0.210			
Mannitol + glycerol	R20_1	0.156	0.158	0.002	1.4
Mannitol + glycerol	R21_2	0.160			

Table S6. Specific growth rates (µmax) measured from CO2 output during the batch growth phase for NBC01270-PTA-kasOp* in the ambr®250 system. Data based on integrated CTR (mmol L^{-1} h^{-1}) values.

Condition	Replicate	µmax (h^{-1})	µmax (mean, h^{-1})	± SD	CV (%)
Mannitol	R18_1	0.085	0.119	0.034	28.5
Mannitol	R19_2	0.153			
Mannitol + glycerol	R22_1	0.230	0.217	0.013	5.9
Mannitol + glycerol	R23_2	0.204			

Supplementary Figures

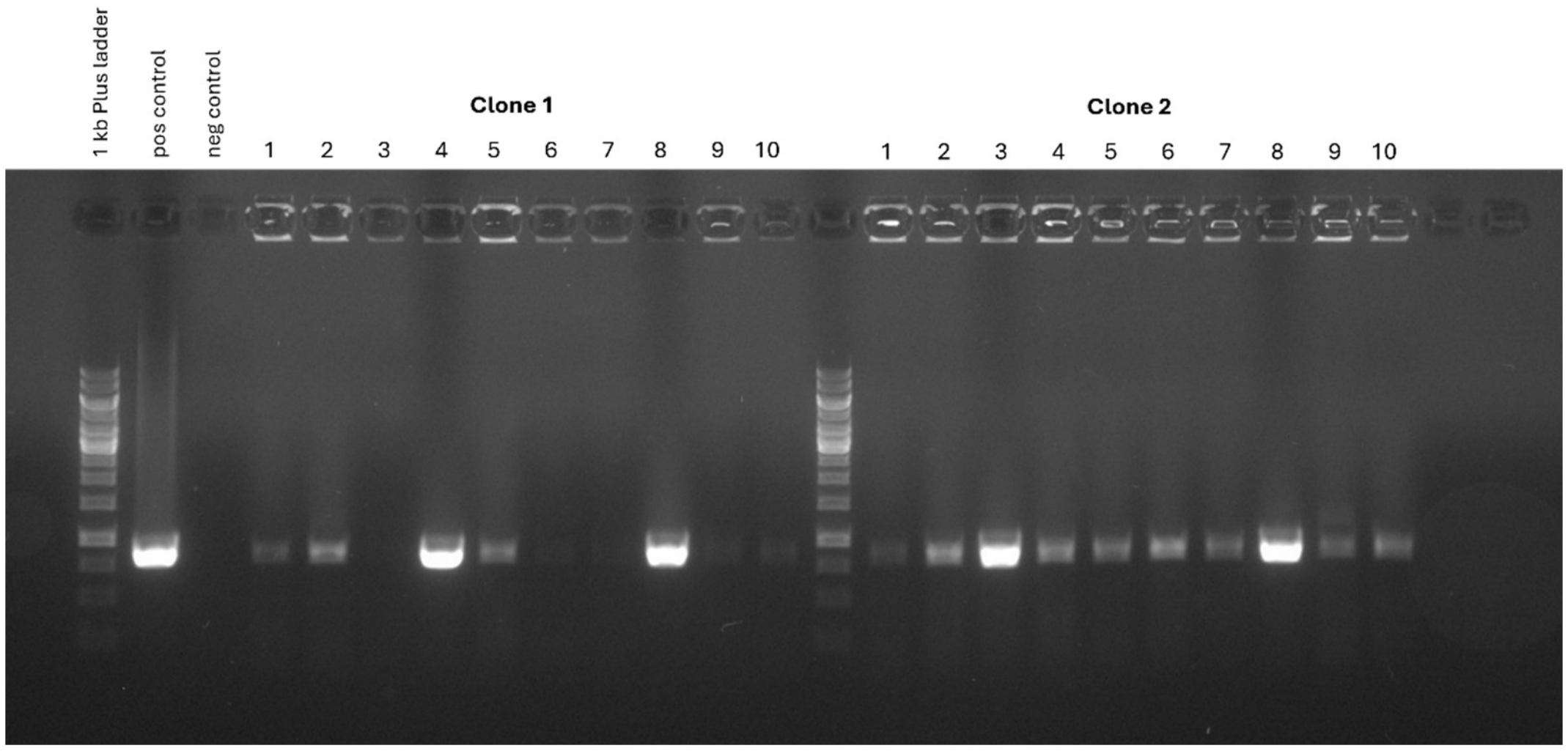

a.

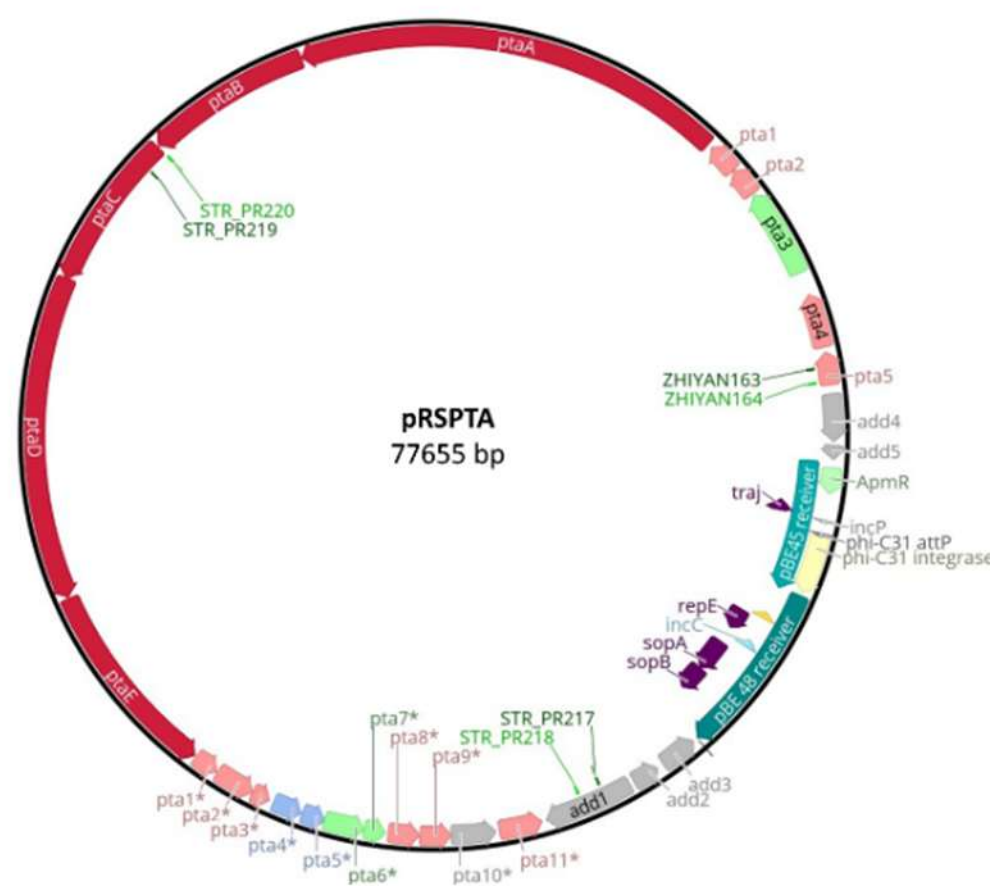

b.

Figure S1: ***Confirmation of cloned BGC using CAPTURE.*** **a.** Colony PCR screening for the center region of the target BGC. 10 white colonies were randomly selected from selective agar plates for clone 1 and 2. Columns 1 – 10: samples/colonies; pos control: genomic DNA of *Streptomyces iranensis* as template; neg control: no genomic DNA added to the PCR reaction; 1 kb Plus DNA ladder (Invitrogen™). **b.** Plasmid map of pRSPTA.

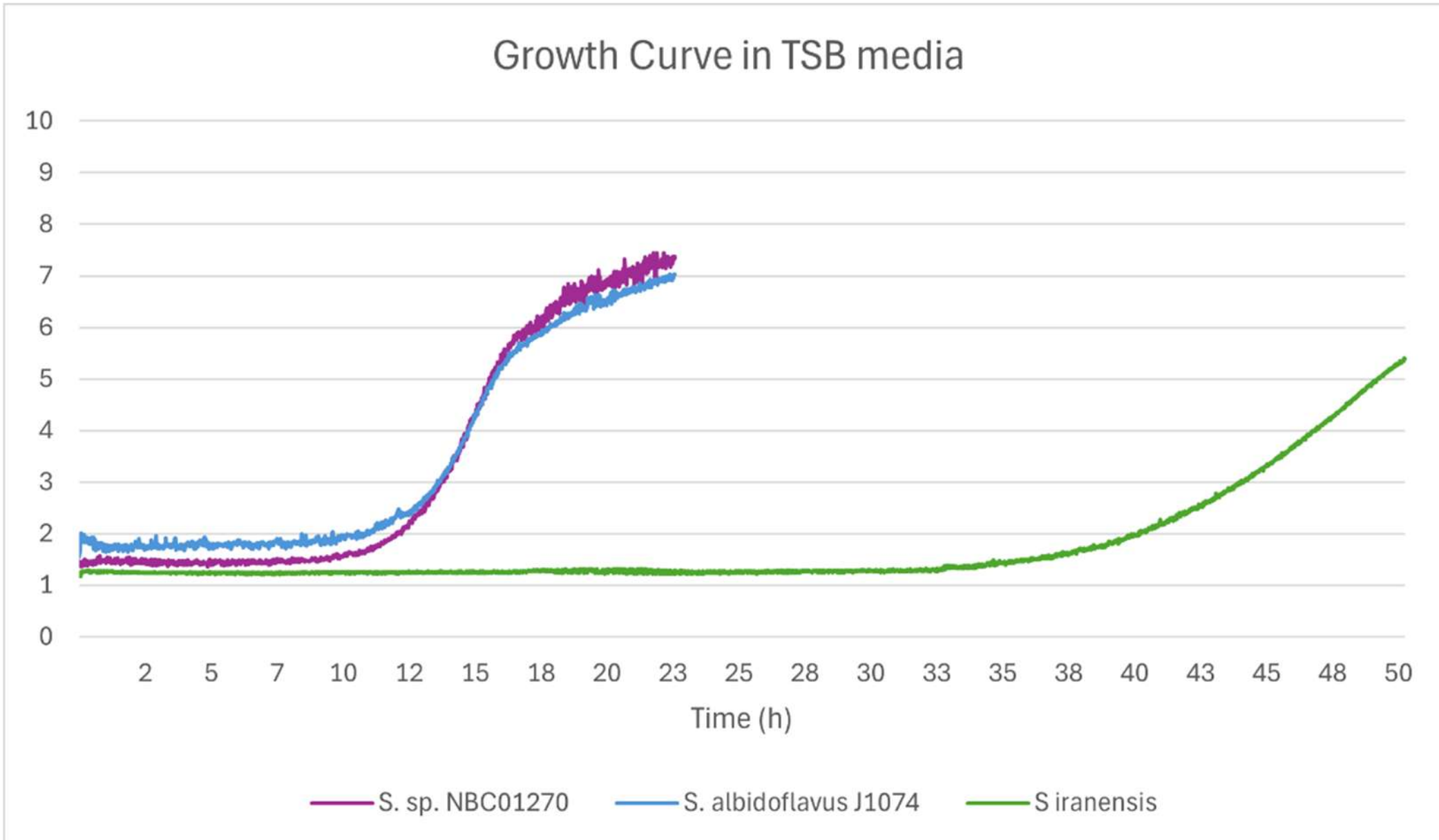

Figure S2: ***Bacterial Growth Curve in TSB Media.*** Growth comparison between heterologous hosts (*S. albidoflavus* J1074 – blue and NBC_01270 – magenta) and native strain (*S. iranensis* – green) in preculture media (TSB). Strains were cultivated in Chi.Bio reactor at 30 °C, 23 or 50 hours. Data points for NBC01270 and *S. albidoflavus* J1074 are absent after 23 hours due to culture termination.

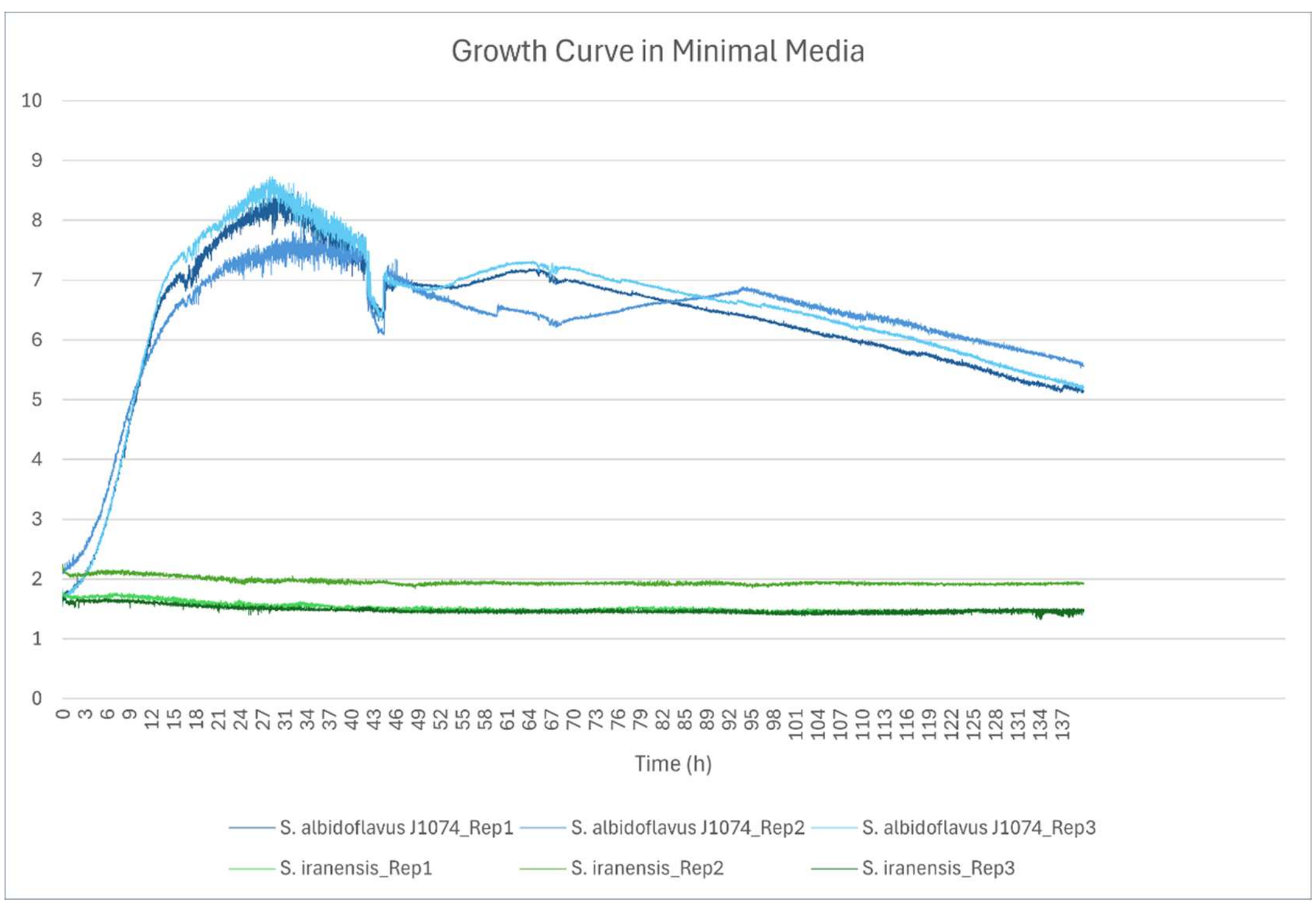

Figure S3: ***Bacterial Growth Curve in Minimal Media.*** Growth comparison between heterologous host (*S. albidoflavus* J1074 – blue) and native strain (*S. iranensis* – green) in minimal media. Strains were cultivated in Chi.Bio reactor at 30 °C, 140 hours.

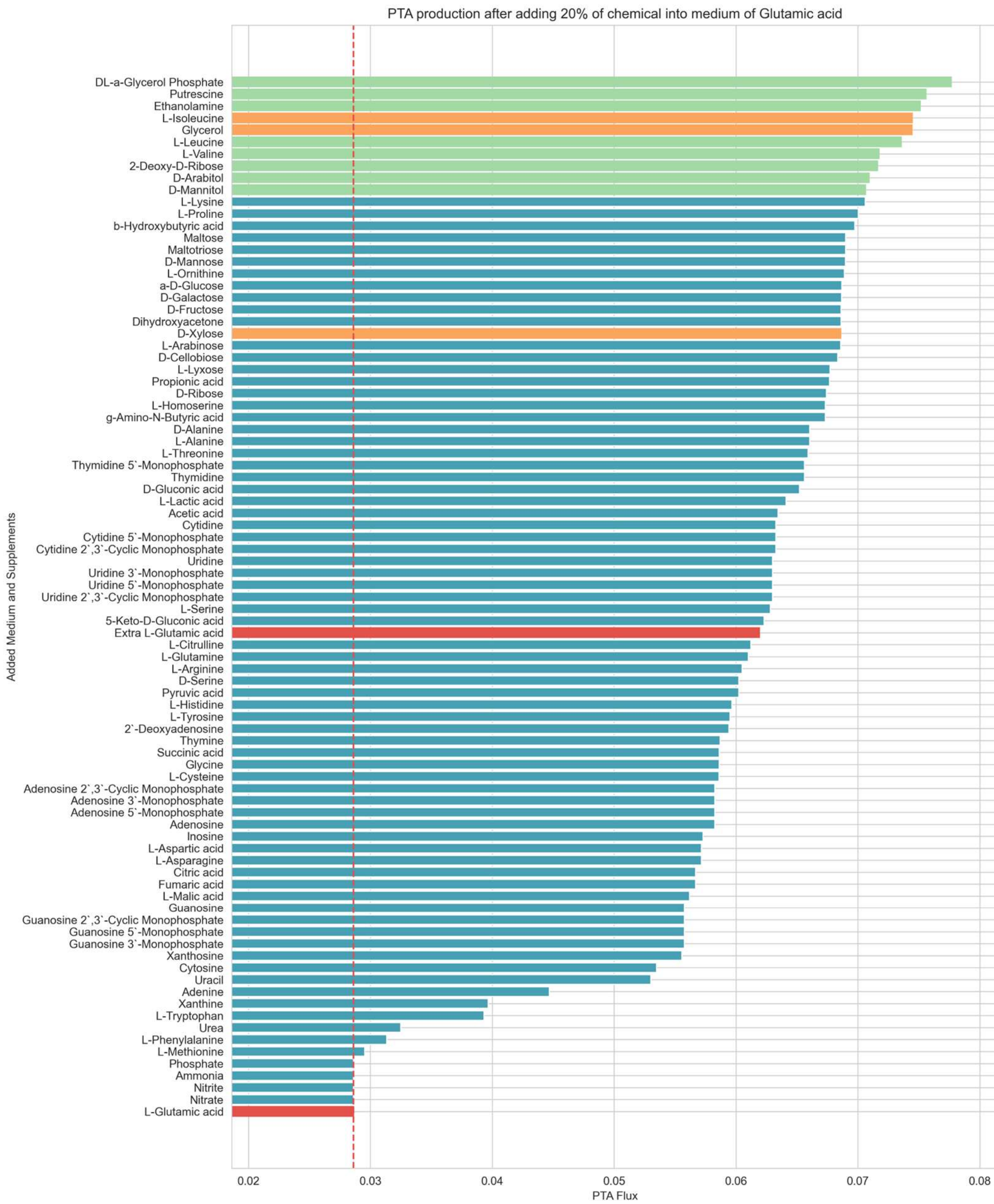
PTA production after adding 20% of chemical into medium of Glutamic acid
Added Medium and Supplements
PTA Flux
0.02
0.03
0.04
0.05
0.06
0.07
0.08
DL-a-Glycerol Phosphate
Putrescine
Ethanolamine
L-Isoleucine
Glycerol
L-Leucine
L-Valine
2-Deoxy-D-Ribose
D-Arabitol
D-Mannitol
L-Lysine
L-Proline
b-Hydroxybutyric acid
Maltose
Maltotriose
D-Mannose
L-Ornithine
a-D-Glucose
D-Galactose
D-Fructose
Dihydroxyacetone
D-Xylose
L-Arabinose
D-Cellobiose
L-Lyxose
Propionic acid
D-Ribose
L-Homoserine
g-Amino-N-Butyric acid
D-Alanine
L-Alanine
L-Threonine
Thymidine 5`-Monophosphate
Thymidine
D-Gluconic acid
L-Lactic acid
Acetic acid
Cytidine
Cytidine 5`-Monophosphate
Cytidine 2`,3`-Cyclic Monophosphate
Uridine
Uridine 3`-Monophosphate
Uridine 5`-Monophosphate
Uridine 2`,3`-Cyclic Monophosphate
L-Serine
5-Keto-D-Gluconic acid
Extra L-Glutamic acid
L-Citrulline
L-Glutamine
L-Arginine
D-Serine
Pyruvic acid
L-Histidine
L-Tyrosine
2`-Deoxyadenosine
Thymine
Succinic acid
Glycine
L-Cysteine
Adenosine 2`,3`-Cyclic Monophosphate
Adenosine 3`-Monophosphate
Adenosine 5`-Monophosphate
Adenosine
Inosine
L-Aspartic acid
L-Asparagine
Citric acid
Fumaric acid
L-Malic acid
Guanosine
Guanosine 2`,3`-Cyclic Monophosphate
Guanosine 5`-Monophosphate
Guanosine 3`-Monophosphate
Xanthosine
Cytosine
Uracil
Adenine
Xanthine
L-Tryptophan
Urea
L-Phenylalanine
L-Methionine
Phosphate
Ammonia
Nitrite
Nitrate
L-Glutamic acid

a.

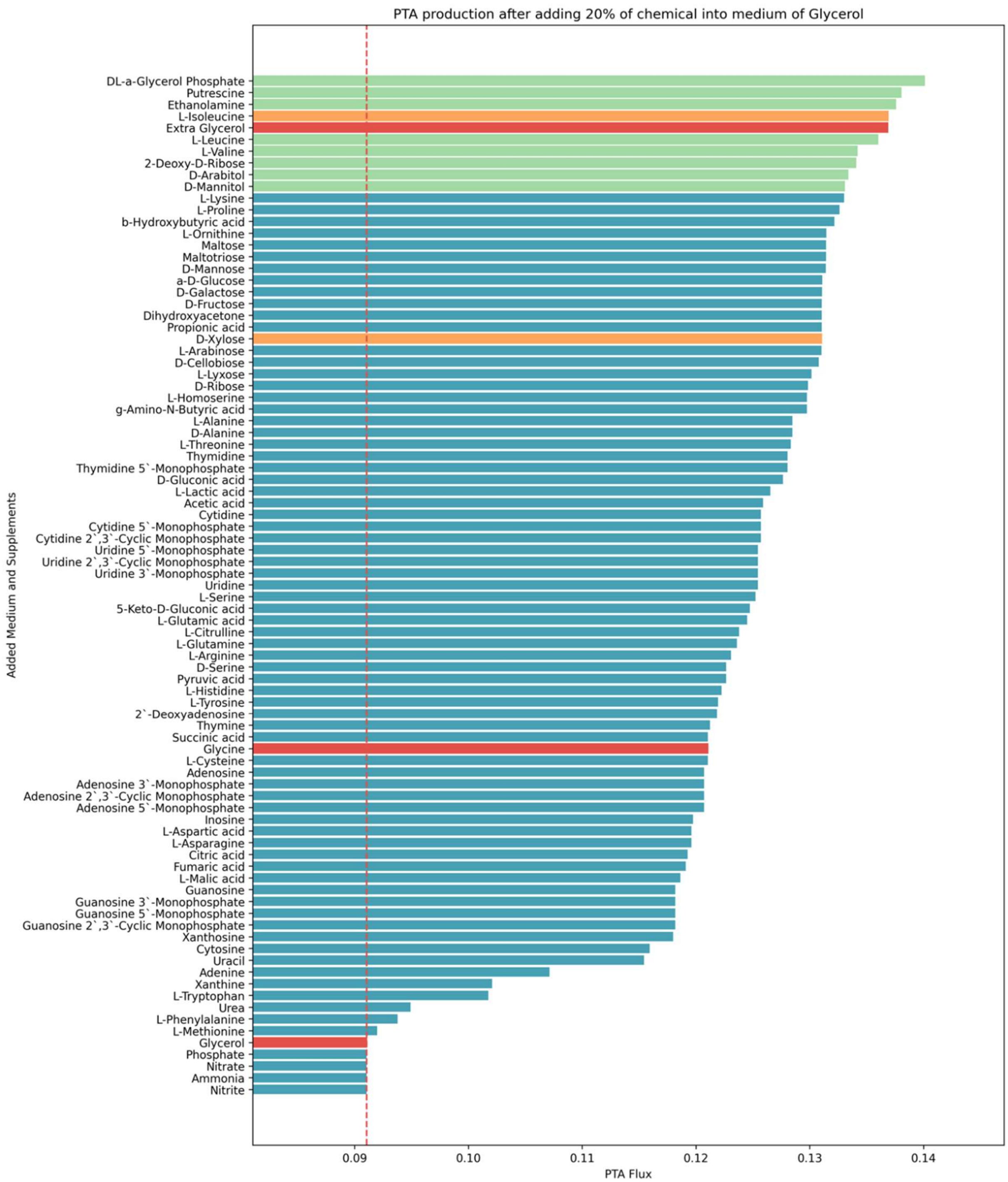
PTA production after adding 20% of chemical into medium of Glycerol
Added Medium and Supplements
DL-a-Glycerol Phosphate
Putrescine
Ethanolamine
L-Isoleucine
Extra Glycerol
L-Leucine
L-Valine
2-Deoxy-D-Ribose
D-Arabitol
D-Mannitol
L-Lysine
L-Proline
b-Hydroxybutyric acid
L-Ornithine
Maltose
Maltotriose
D-Mannose
a-D-Glucose
D-Galactose
D-Fructose
Dihydroxyacetone
Propionic acid
D-Xylose
L-Arabinose
D-Cellobiose
L-Lyxose
D-Ribose
L-Homoserine
g-Amino-N-Butyric acid
L-Alanine
D-Alanine
L-Threonine
Thymidine
Thymidine 5`-Monophosphate
D-Gluconic acid
L-Lactic acid
Acetic acid
Cytidine
Cytidine 5`-Monophosphate
Cytidine 2`,3`-Cyclic Monophosphate
Uridine 5`-Monophosphate
Uridine 2`,3`-Cyclic Monophosphate
Uridine 3`-Monophosphate
Uridine
L-Serine
5-Keto-D-Gluconic acid
L-Glutamic acid
L-Citrulline
L-Glutamine
L-Arginine
D-Serine
Pyruvic acid
L-Histidine
L-Tyrosine
2`-Deoxyadenosine
Thymine
Succinic acid
Glycine
L-Cysteine
Adenosine
Adenosine 3`-Monophosphate
Adenosine 2`,3`-Cyclic Monophosphate
Adenosine 5`-Monophosphate
Inosine
L-Aspartic acid
L-Asparagine
Citric acid
Fumaric acid
L-Malic acid
Guanosine
Guanosine 3`-Monophosphate
Guanosine 5`-Monophosphate
Guanosine 2`,3`-Cyclic Monophosphate
Xanthosine
Cytosine
Uracil
Adenine
Xanthine
L-Tryptophan
Urea
L-Phenylalanine
L-Methionine
Glycerol
Phosphate
Nitrate
Ammonia
Nitrite
0.09
0.10
0.11
0.12
0.13
0.14
PTA Flux

b.

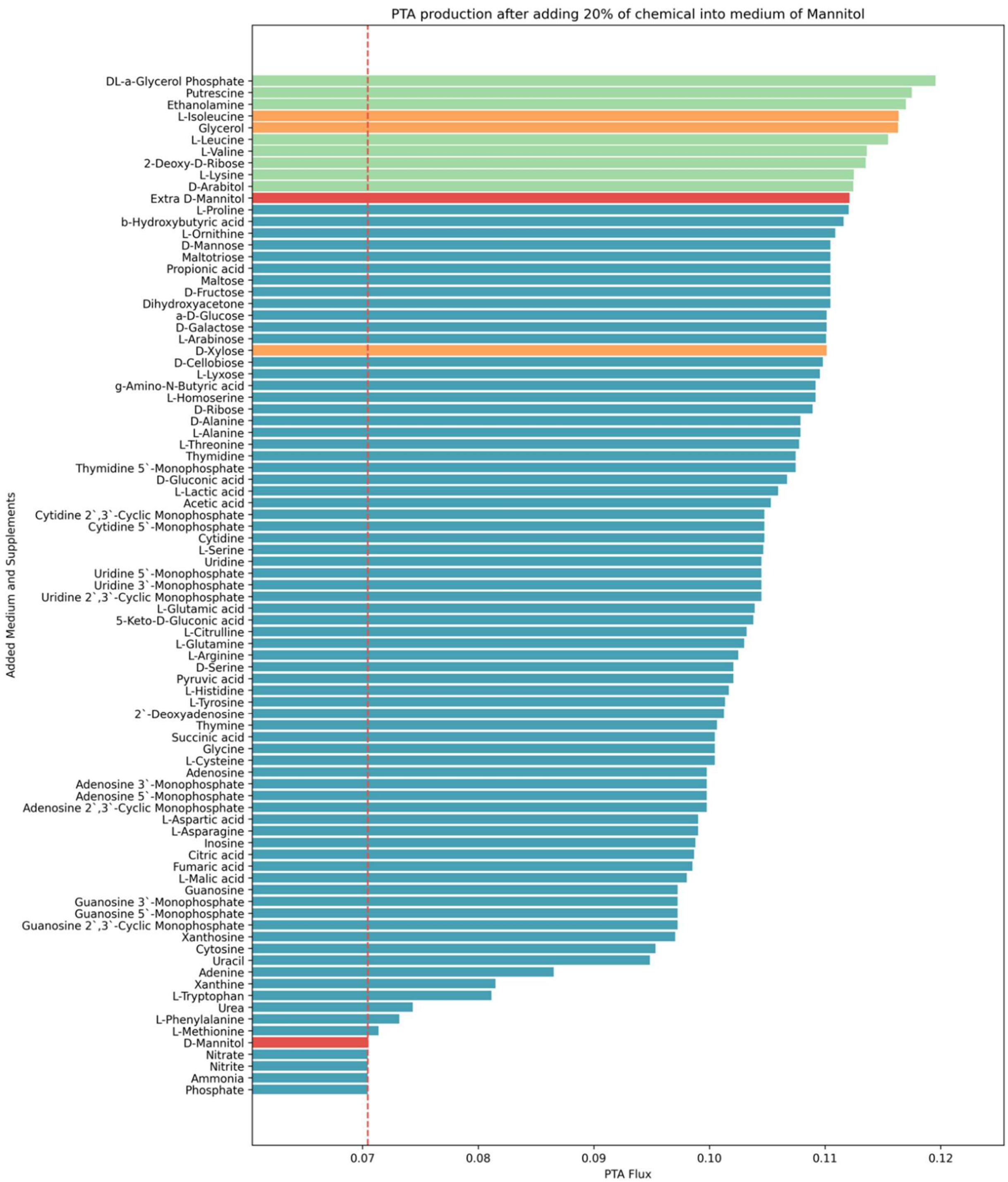

c.

Figure S4: ***Genome-scale model predictions of the effect of individual supplements on PTA production in a. glutamate-, b. glycerol-, and c. mannitol-based media*.** The horizontal bars represent the predicted effects of different supplements on PTA production based on *in silico* simulation, relative to the baseline level achieved with the non-supplemented medium (indicated by the dashed vertical line). Red vertical bars represent the original base medium and a positive control containing 20% additional base carbon source (e.g., "Extra D-mannitol"). Green vertical bars indicate the top 10-ranked supplements predicted to improve PTA production, while orange vertical bars highlight the compounds selected for experimental validation in this study.

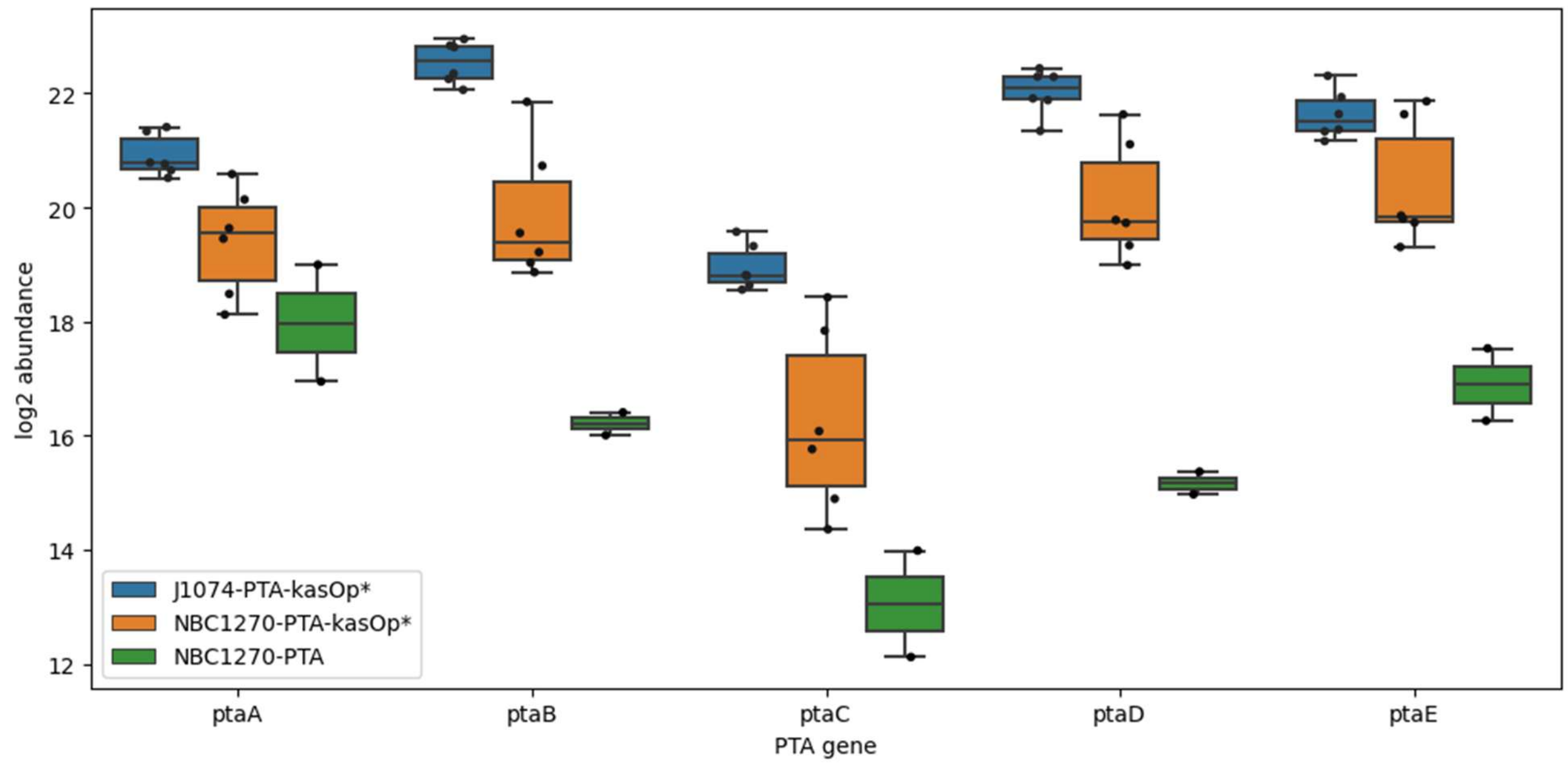

Figure S5: ***Proteomic detection of PTA BGC-associated proteins in heterologous strains under productive cultivation conditions.*** Protein abundance levels of PtaA–PtaE are shown as log2 abundance for J1074-PTA-*kasO*p* (blue), NBC1270-PTA-*kasO*p* (orange), and NBC1270-PTA (green). Promoter engineered strains were analyzed across MM, Am2ab, and DNPM media, while NBC1270-PTA was analyzed from MS-liquid medium, corresponding to conditions where PTA- or elaiophylin were detected. Boxplots represent the distribution of protein abundance across media conditions, and dots indicate individual biological replicates. These data provide qualitative evidence of PTA BGC expression at the protein level under productive cultivation conditions.

References

Acin-Albiac M, Filannino P, Gobbetti M, Di Cagno R (2020) Microbial high throughput phenomics: The potential of an irreplaceable omics. Comput Struct Biotechnol J 18:2290–2299. https://doi.org/10.1016/j.csbj.2020.08.010

Bierman M, Logan R, O'Brien K, Seno ET, Nagaraja Rao R, Schoner BE (1992) Plasmid cloning vectors for the conjugal transfer of DNA from *Escherichia coli* to *Streptomyces* spp. Gene 116:43–49. https://doi.org/10.1016/0378-1119(92)90627-2

Callister SJ, Barry RC, Adkins JN, Johnson ET, Qian WJ, Webb-Robertson BJM, Smith RD, Lipton MS (2006) Normalization Approaches for Removing Systematic Biases Associated with Mass Spectrometry and Label-Free Proteomics. J Proteome Res 5:277–286. https://doi.org/10.1021/PR050300L

Galardini M, Mengoni A, Biondi EG, Semeraro R, Florio A, Bazzicalupo M, Benedetti A, Mocali S (2014) DuctApe: A suite for the analysis and correlation of genomic and OmniLog™ Phenotype Microarray data. Genomics 103:1–10. https://doi.org/10.1016/j.ygeno.2013.11.005

Hwang K-S, Kim HU, Charusanti P, Palsson BØ, Lee SY (2014) Systems biology and biotechnology of *Streptomyces* species for the production of secondary metabolites. Biotechnol Adv 32:255–268. https://doi.org/10.1016/j.biotechadv.2013.10.008

Jönsson M, Sigrist R, Gren T, Semenov Petrov M, Marcussen NEJ, Svetlova A, Charusanti P, Gockel P, Palsson BO, Yang L, Özdemir E (2025) Machine learning uncovers the transcriptional regulatory network for the production host *Streptomyces albidoflavus*. Cell Rep 44:115392. https://doi.org/10.1016/j.celrep.2025.115392

Jørgensen TS, Mohite OS, Sterndorff EB, Alvarez-Arevalo M, Blin K, Booth TJ, Charusanti P, Faurdal D, Hansen TØ, Nuhamunada M, Mourched A-S, Palsson BØ, Weber T (2024) A treasure trove of 1034 actinomycete genomes. Nucleic Acids Res 52:7487–7503. https://doi.org/10.1093/nar/gkae523

Kittikunapong C, Ye S, Magadán-Corpas P, Pérez-Valero Á, Villar CJ, Lombó F, Kerkhoven EJ (2021) Reconstruction of a Genome-Scale Metabolic Model of *Streptomyces albus* J1074: Improved Engineering Strategies in Natural Product Synthesis. Metabolites 11:304. https://doi.org/10.3390/metabo11050304

MacNeil DJ, Occi JL, Gewain KM, MacNeil T, Gibbons PH, Ruby CL, Danis SJ (1992) Complex organization of the *Streptomyces avermitilis* genes encoding the avermectin polyketide synthase. Gene 115:119–125. https://doi.org/10.1016/0378-1119(92)90549-5

Tong Y, Charusanti P, Zhang L, Weber T, Lee SY (2015) CRISPR-Cas9 Based Engineering of Actinomycetal Genomes. ACS Synth Biol 4:1020–1029. https://doi.org/10.1021/acssynbio.5b00038

Zaburannyi N, Rabyk M, Ostash B, Fedorenko V, Luzhetskyy A (2014) Insights into naturally minimised *Streptomyces albus* J1074 genome. BMC Genomics 15:97. https://doi.org/10.1186/1471-2164-15-97
